# Multicomponent Lifestyle Interventions for Treating Overweight and Obesity in Children and Adolescents: A Systematic Review and Meta-Analyses

**DOI:** 10.1155/2017/5021902

**Published:** 2017-12-17

**Authors:** I. K. Ø. Elvsaas, L. Giske, B. Fure, L. K. Juvet

**Affiliations:** ^1^Norwegian Institute of Public Health, Oslo, Norway; ^2^The Arctic University of Norway, Tromsø, Norway; ^3^University College of Southeast Norway, Notodden, Norway

## Abstract

**Background:**

Treatment of childhood obesity is important in preventing development of obesity-related diseases later in life. This systematic review evaluates the effect of multicomponent lifestyle interventions for children and adolescents from 2 to 18 years.

**Methods and Results:**

We performed systematic searches in nine databases. Thirty-nine studies met the criteria for meta-analyses. We found a significant difference in body mass index (BMI) after 6 months (MD −0.99 (95% CI −1.36 to −0.61)), 12 months (MD −0.67 (95% CI −1.01 to −0.32)), and 24 months (MD −0.96 (95% CI −1.63 to −0.29)) in favour of multicomponent lifestyle interventions compared to standard, minimal, and no treatment. We also found a significant difference in BMI *Z* scores after 6 months (MD −0.12 (95% CI −0.17 to −0.06)), 12 months (MD −0.16 (95% CI −0.21 to −0.11)), and 24 months (MD −0.16 (95% CI −0.21 to −0.10)) in favour of multicomponent lifestyle interventions. Subgroup analyses suggested an increased effect in specialist health care with a group treatment component included in the intervention.

**Conclusion:**

Multicomponent lifestyle interventions have a moderate effect on change in BMI and BMI *Z* score after 6, 12, and 24 months compared with standard, minimal, and no treatment.

## 1. Background

The prevalence of overweight and obesity among children and adolescents has risen in the past decades [1]. In Norway, 14% of children and adolescents are overweight or obese [2]. According to the Centers for Disease Control and Prevention [3], overweight is defined as a body mass index (BMI) between the 85th and 95th percentile range and obesity as a BMI at or above the 95th percentile for children and adolescents of the same age and gender.

Overweight and obesity can negatively affect physical and possibly psychological health and are associated with accumulation of cardiovascular risk factors [4] and risk of type 2 diabetes mellitus [5]. Obesity in adolescents increases the risk of severe obesity in adulthood [6] and may cause morbidity and early mortality [7, 8]. Thus, early detection and treatment may lead to major health benefits.

Overweight and obesity arise from energy imbalance. Reasons for this energy imbalance are multifactorial and include unhealthy eating patterns, lack of physical activity and excessive inactivity, genetic factors, and social structures [9–11]. Because of individual variation, some groups and individuals are more vulnerable than others.

Multicomponent lifestyle interventions that include behavioural interventions to alter dietary habits and increase physical activity are commonly used [12, 13] and are the preferred methods to treat overweight and obesity in children and adolescents [14]. BMI and BMI standard deviation scores (*Z* scores) are regularly used to assess effectiveness of lifestyle interventions. BMI *Z* scores indicate how many standard deviations children's BMI is above or below the average BMI value for their age group and gender [15] in a given reference population. BMI *Z* scores seem to be acceptable for assessing overweight in children and adolescents aged 2 to 19 years [16]. However, the BMI *Z* score has limitations [17], and for obesity, BMI may be a more useful measurement [18]. Commonly, multicomponent lifestyle interventions have produced losses of 5 to 20 percent of excess weight, or 1 to 3 BMI units over 3 to 6 months in children [19]. Over 6 to 12 months, the change has ranged from 25 percent loss to 10 percent increase in excess weight, or 0 to 4 BMI units [19].

Recently, a series of Cochrane reviews on diet, physical activity, and behavioural interventions compared to control conditions were published [20–22]. For children up to the age of 6 years, the reviews found a reduction in BMI *Z* scores up to 2-year follow-up in favour of diet, physical activity, and behavioural interventions [20]. The reviews also found a reduction in BMI and BMI *Z* scores for both children and adolescents aged 6 to 17 years [21, 22] in analyses with the longest follow-up data, for at least 6 months, in favour of diet, physical activity, and behavioural interventions.

The aim of this systematic review is to assess the effect of multicomponent lifestyle interventions including two or more lifestyle components on change in BMI and BMI *Z* scores in children (2 to <12 years) and adolescents (≥12 to 18 years) compared to control conditions of standard, minimal, or no treatment at 6-, 12-, and 24-month follow-up. We distinguish between 6-, 12-, and 24-month follow-up data, in order to illustrate effect estimates at various follow-ups.

## 2. Material and Methods

### 2.1. Literature Search and Selection

The review was performed according to Preferred Reporting Items for Systematic reviews and Meta-Analysis (PRISMA) [23]. We performed systematic literature searches in the Cochrane Database of Systematic Reviews, Cochrane Central Register of Controlled Trials (CENTRAL), Medline (Ovid), Embase (Ovid), CINAHL via EBSCOhost, PsycINFO, ISI Web of Science, DARE (Database of Abstracts of Reviews of Effects), and HTA. To avoid duplication, we first searched for systematic reviews up to June 2012. After a reviewing process, we found one systematic review [24] with systematic searches up to May 2008 that met our criteria. We then searched for RCTs from January 2008 to February 2015. The search strategies were adapted from the search strategies in the identified systematic review [24] to each database and based on MeSH terms and keywords such as “overweight,” “obesity,” “body mass index,” “child,” and “adolescents” with synonyms. The complete search strategies have been published previously [25].

Two researchers independently reviewed abstracts and full-text articles in two steps: first for systematic reviews of RCTs and then for RCTs. Finally, RCTs were included if they included children (2 to 18 years) who are overweight or obese, assessed effects of multicomponent lifestyle interventions (consisting of at least two strategies on altering diet, physical activity, and behaviour), assessed BMI and/or BMI *Z* score from baseline to 6, 12, and/or 24 months, and used principles of intention-to-treat analyses or had no loss to follow-up. Comparisons were standard, minimal, or no treatment. Targets of the interventions were children and adolescents with or without family involvement. The interventions could take place in schools, primary care, hospitals, or other health institutions. Exclusion criteria were children younger than two years, type 1 diabetes mellitus, secondary or syndromic cause of obesity, and participant pregnancy. Discrepancies between reviewers about inclusion and/or exclusion were resolved by consulting one of the coauthors of the paper.

### 2.2. Quality Assessment

Two reviewers independently assessed the risk of bias according to the Cochrane Handbook for Systematic Reviews of Interventions [26]. The following criteria were evaluated: (a) random sequence generation, (b) allocation concealment, (c) blinding of participants and personnel, (d) blinding of outcome assessment, (e) incomplete outcome data, (f) selective reporting, and (g) other sources of bias. We judged the risk of bias as “low risk,” “unclear risk,” and “high risk.” We used the Grading of Recommendations, Assessment, Development, and Evaluation (GRADE) [27] to assess the quality of the overall documentation from pooled results in meta-analyses. Our confidence in the outcome results was rated as high, moderate, low, or very low based on assessment of five domains of the evidence (risk of bias, indirectness, imprecision, inconsistency, and reporting bias).

### 2.3. Data Extraction and Statistical Analyses

One reviewer extracted data on every included study, and another reviewer controlled the data. We extracted the first author's last name, publication year, intervention type, duration and follow-up, comparison, location where the study was performed, sample size, BMI and BMI *Z* score, or change in BMI and BMI *Z* score at 6-, 12-, and/or 24-month follow-up.

Meta-analyses were performed using the software Review Manager 5.3. We used a “random effects” model that takes into account potential differences between studies. We expressed continuous data as mean difference (MD) with 95% confidence interval (CI). Heterogeneity between studies was tested with *I*-Square (*I*^2^), where a high value (*I*^2^ > 50–60%, *P* value ≤ 0.1) indicates statistically significant heterogeneity between studies. High statistical heterogeneity will affect our confidence in the overall results. We tested publication bias graphically using “funnel plots” and used this information in the GRADE assessment.

Outcome data were BMI and BMI *Z* score of multicomponent interventions compared to standard, minimal, or no intervention at 6, 12, and 24 months. According to the Cochrane Handbook for Systematic Reviews of Interventions [26], we included both change scores and final scores in the meta-analyses. Change score was preferred, and we used final score only where change scores were not available. In studies with more than two study arms, only the most intensive intervention compared to minimal or no control condition was included in meta-analyses. We conducted subgroup analyses for children (<12 years) and adolescents (≥12 years) and subgroup analyses to explore heterogeneity based on the control groups. We also conducted subgroup analyses based on treatment setting and treatment organization.

## 3. Results

### 3.1. Search Results and Selection of Studies

**Figure 1:**
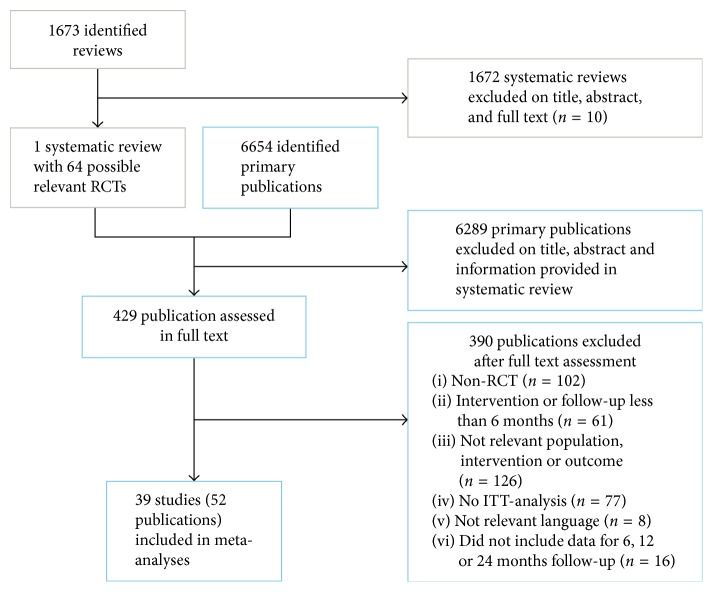
Flow diagram.

Our database searches for reviews from January 2007 to June 2012 retrieved 1673 references, and our search for clinical trials from January 2008 to February 2015 retrieved 6654 references. We included one systematic review [24] with final search date of May 2008, from which we identified seven RCTs. To identify newer RCTs, we searched from 2008 to February 2015 and identified 32 additional RCTs. In total, we included 39 RCTs in 52 publications [28–79] with data that could be included in meta-analyses on the effect of multicomponent interventions on change in BMI and/or BMI *Z* scores.  Figure 1 shows the flow diagram of the search process and selection of studies.

### 3.2. Risk of Bias

**Figure 2:**
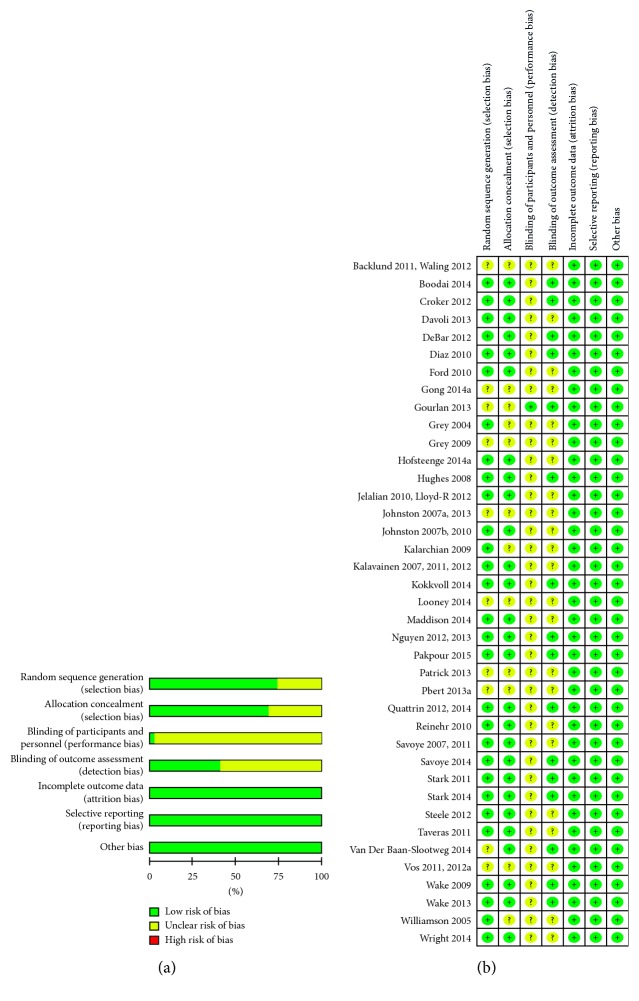
(a) Risk of bias overall diagram; (b) risk of bias individual study.

Risk of bias in included studies (*n* = 39) is provided in Figures 2 and 2. Fourteen studies were judged to have low risk of bias in all categories except for blinding of participants and personnel. Furthermore, we judged 12 studies to have low risk of bias in all categories except for blinding of participants and personnel and blinding of outcome assessors. In 12 studies, random sequence generation and/or allocation concealment and blinding of participants, personnel, and assessors were judged to have unclear risk of bias. One study was judged to have unclear risk of bias regarding random sequence generation and allocation concealment but low risk of bias in all other categories. Because BMI and BMI *Z* score are objective measures, we chose not to increase the risk of bias for lack of blinding.

### 3.3. Description of Included Studies

Detailed characteristics of the 39 RCTs (*n* = 20 for mean < 12 years and *n* = 19 for mean ≥ 12 years) included in meta-analyses are presented in Supplementary Tables 1 and 2 available online at https://doi.org/10.1155/2017/5021902. The included studies were conducted in North America (*n* = 20), Europe (*n* = 12), Oceania (*n* = 4), the Middle East (*n* = 2), and Asia (*n* = 1). In total, there were 5,397 participants, aged 2 to 18 years. Individual study populations ranged from 18 to 475 participants. The interventions lasted from 10 weeks to 24 months, but most studies had interventions that lasted for 6 (*n* = 16) or 12 (*n* = 12) months. Thirty-five of the studies had two study arms, three had three arms, and one had four arms.

The interventions consisted of two or more of the following: increase in physical activity, reduction of sedentary activity, and change in dietary habits and behavioural strategies, including motivational interviewing. Thirty-two studies [28–33, 35, 36, 40–56, 58, 59, 61–71, 73–79] included interventions for change in behaviour, dietary habits, and physical activity/sedentary activity levels. One study included change in behaviour and in physical activity levels [57]. Three studies [34, 60, 72] assessed the effect of motivational interviews on change in both dietary habits and physical activity levels, and one study [39] assessed the effect of motivational interviews on the physical activity level. One study [38] assessed mainly change in dietary habits but also focused on change in eating behaviour. One study [37] assessed the effect of a method to change eating behaviour in combination with family-based lifestyle intervention.

The interventions were conducted in specialist health care (*n* = 19), primary health care (*n* = 11), combination of specialist and primary health care (*n* = 1), schools (*n* = 7), or via Internet (*n* = 1). In most of the RCTs (*n* = 37), interventions were directed to the whole family, to the child/adolescent and at least one caregiver, or to the child/adolescent and parents in separate meetings, and in two cases, interventions were directed directly to the child/adolescent with written consent from parents or caregiver. Comparisons were waiting list or no intervention (*n* = 8), standard care (*n* = 20), and minimal intervention or self-help (*n* = 11). The studies included children and adolescents who are overweight alone (≥85 percentile to ≥95 percentile, *n* = 1), both overweight and obese (≥85 percentile, *n* = 21), and obese alone (≥95 percentile, *n* = 17). Some differences existed, however, in definitions of overweight and obesity (see Supplementary Table 1).

We aimed to include studies using ITT analyses to compensate for loss to follow-up. The methods for replacing missing values were inadequately described in many of the included studies (see Supplementary Table 1), but both baseline carried forward and last observation carried forward, as well as multiple methods for imputing missing data, were used. Dropout occurred in 36 studies and varied from <3 to 48% from intervention start to end. Loss to follow-up (after the end of intervention) varied from <3 to 57%. Registered reasons for dropout and loss to follow-up included that the participants did not want to continue, did not meet for follow-up, and had moved, family problems, long journey or problems with transportation, time conflict, and illness or injury not related to the intervention. Three studies with no dropout were included in meta-analyses due to low risk of attrition bias.

### 3.4. Change in BMI and BMI *Z* Scores

**Figure 3:**
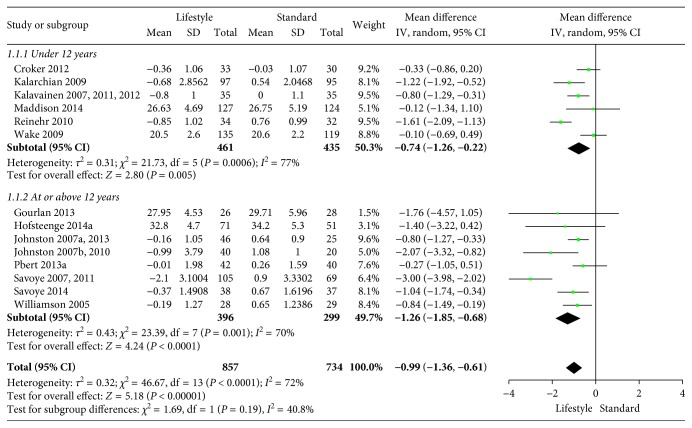
BMI at 6-month follow-up.

**Figure 4:**
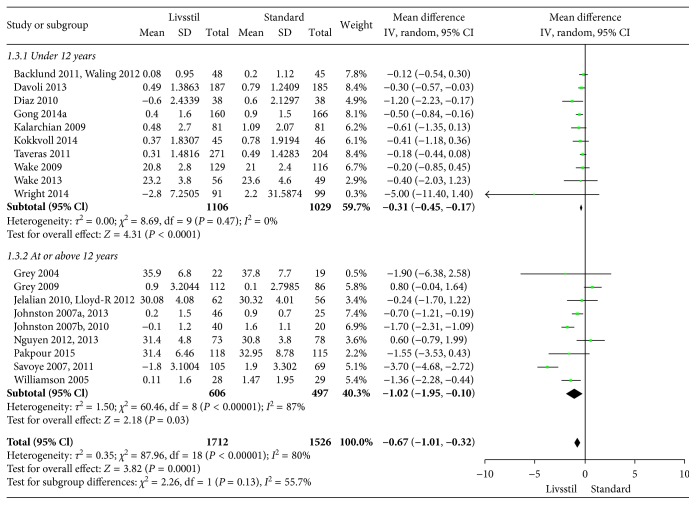
BMI at 12-month follow-up.

**Figure 5:**
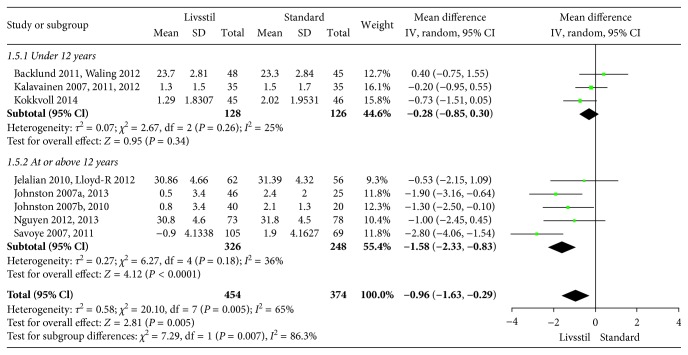
BMI at 24-month follow-up.

**Figure 6:**
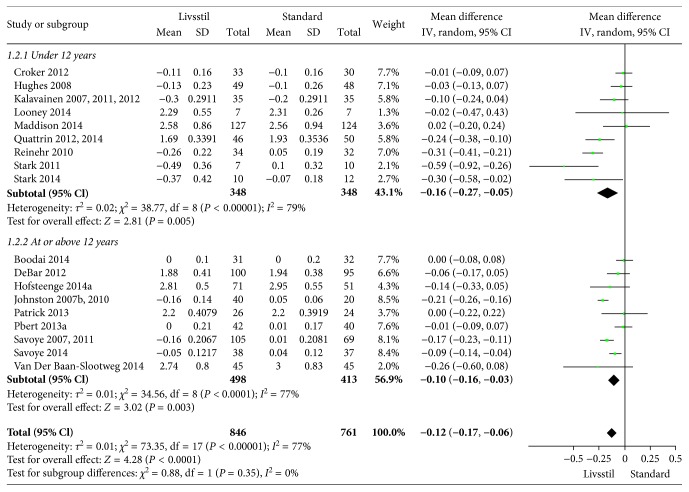
BMI *Z* scores at 6-month follow-up.

**Figure 7:**
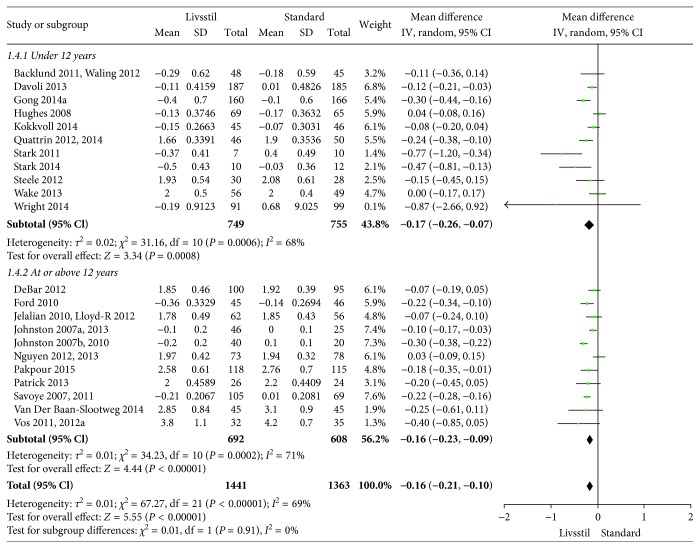
BMI *Z* scores at 12-month follow-up.

**Figure 8:**
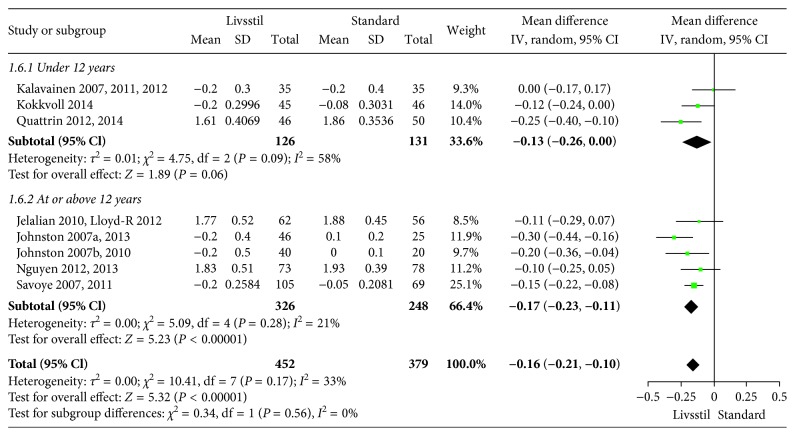
BMI *Z* scores at 24-month follow-up.

The included interventions were judged to be sufficiently similar to be pooled in meta-analyses and differentiated in meta-analyses for follow-up data at 6, 12, and/or 24 months (Figures 3–8). For BMI, there were 14, 19, and 8 studies, respectively, that included data for meta-analyses at 6-, 12-, and 24-month follow-up. For BMI *Z* score, there were 18, 22, and 11 studies, respectively, that included data for meta-analyses at 6-, 12-, and 24-month follow-up. The information regarding calculation of the BMI *Z* scores in the studies was somewhat limited (see Supplementary Table 1). All main analyses showed significant differences in BMI and BMI *Z* scores in favour of interventions compared with control conditions (Table 1).

In subgroup analyses for children under 12 years of age and adolescents of 12 years of age or older (Table 1), we found that the intervention effect for BMI was somewhat larger for adolescents compared to children. However, the difference was only statistically significant at 24-month follow-up. For BMI *Z* scores, there were no statistical differences in subgroup analyses between children and adolescents at any follow-up point.

### 3.5. Quality of the Overall Documentation

We judged the overall quality of the pooled estimates for multicomponent lifestyle interventions compared with standard, minimal, and no intervention with GRADEpro GDT [27] (Supplementary Tables 3(a) and 3(b)). We have moderate confidence in the effect estimate for change in BMI and BMI *Z* score at 6- and 12-month follow-up. We have low confidence in the effect estimate for change in BMI at 24-month follow-up and high confidence in the effect estimate for change in BMI *Z* scores at 24-month follow-up. The main reason for downgrading the overall quality was high statistical heterogeneity (inconsistency) in the meta-analyses. We suspected that the high statistical heterogeneity was due to differences in comparison groups, but exploratory subgroup analyses on different comparisons (standard, minimal, or no intervention) failed to explain the observed heterogeneity (*I*^2^ from zero to 91%, data available at [25]).

## 4. Subgroup Analyses on Treatment Setting and Organization

We performed two additional subgroup analyses on change in BMI based on four treatment settings and three types of organization (Table 2). A larger effect of multicomponent lifestyle interventions was observed in specialist health care compared to primary health care- and school-based interventions at 6- and 12-month follow-up, but this was not evident in subgroup analyses at 24 months. We cannot conclude on the effectiveness of Internet counselling because of sparse data. An intervention with a group treatment component suggested increased effect compared to individual treatment at 6-month follow-up, but our analyses could not reveal if this was true also for results at 12 and 24 months.

## 5. Discussion

This systematic review provides evidence for moderate treatment effects of multicomponent interventions on the weight-related outcomes BMI and BMI *Z* scores for children and adolescents who are overweight or obese. Our findings are in accordance with findings in other reviews [13, 20–22, 80–82]. Our results at 6 and 12 months are similar to the lower end of what can be expected after multicomponent interventions [19]. Overall, our review extends the evidence base on use of multicomponent interventions in treatment of childhood overweight and obesity and indicates the treatment effect up to 24-month follow-up. From subgroup analyses, it seems that the most effective interventions are given in specialist healthcare with a group treatment component.

We performed subgroup analyses based on treatment setting (specialist health care, primary health care, schools, and Internet). Our data suggested increased effect in specialist health care at 6- to 12-month follow-up compared to other settings. A possible explanation for this finding may be lack of standardized procedures at the primary care level [83]. Banks and coworkers [84] found that interventions carried out in primary care settings have the potential to be effective in providing weight management for children when a hospital-based obesity management program is offered in a primary care setting. However, more studies are needed to confirm these findings. Other studies find that school-based interventions have the potential to be effective in combating overweight and obesity since many children lack resources, education, and support outside of their homes [85]. Studies have shown that school-based interventions can be effective, especially among older children and adolescents, and when families are included [86]. Due to limited data, we cannot conclude on the effectiveness of Internet interventions, but we suppose that further interventions will be based on Internet and social networking. Today, many adolescents are familiar with using smart phones and other devices, and there is a great opportunity to incorporate technology into intervention delivery [87].

Our subgroup analyses based on treatment organization (group, individual, group and individual) suggested an increased effect with a group treatment component included in the intervention as opposed to individual treatment. Few studies have investigated the differences between effectiveness of group therapy and individual therapy in obesity management among children. However, our findings are supported by one study in which the authors found somewhat larger reduction in weight-related outcomes with group treatment compared to individual treatment [88, 89]. A possible explanation for effectiveness of group interventions can be related to children's positive social experiences such as having fun and making friends that can foster the desire to continue attending [90]. For participants who attend treatment interventions, benefits are often compromised by high programme attrition [90]. High dropout rates may indicate that obesity management is perceived as an optional service, where dropout can be assumed to have little medical consequences [84]. Also, high dropout rates may indicate how satisfied the participants are with the intervention and how achievable it is [80]. Reasons for nonattendance and dropouts have been assessed in several studies [90–92] and include lack of weight loss success and such family barriers as lack of time or logistical barriers, perceived costs of healthy food, lack of exercise options, and unmet family needs.

Attrition may increase the difficulty of determining treatment effectiveness. Our review revealed high dropout rates in most of the included studies. We therefore included studies with ITT analyses to minimize possible bias due to dropouts. Studies without dropout were included in the analyses due to low risk of attrition bias. Family barriers to continued participation in treatment, however, remain a challenge for determining treatment success. Improved study design that takes family barriers into account may contribute to higher attendance rates and larger treatment effects [93].

### 5.1. Strengths and Limitations in the Assessment

We searched systematically in several databases for systematic reviews of RCTs. The evidence base consists of RCTs identified through one high-quality systematic review up to 2008 and through searches for RCTs from 2008 to 2015. We may have missed relevant studies published before 2008 by using a systematic review to identify RCTs. Also, our last search for literature was conducted in February 2015. We cannot therefore rule out that the accuracy of our effect estimates would change with new studies. Nevertheless, Snethen and coworkers calculated, using a fail-safe N model, that it would take 335 unpublished studies that did not demonstrate weight loss in children who are overweight to negate the positive findings in their meta-analysis on the effect of lifestyle interventions [82]. Based on this, we therefore assume that our overall results that multicomponent interventions are effective on BMI and BMI *Z* score reduction in children and adolescents probably will not change unless numerous studies demonstrating no effect on BMI or BMI *Z* score reduction are published.

In our risk of bias judgement, we found that 13 of 39 had inadequate description of randomization and/or allocation concealment. Only one study had made an effort to blind both participants and assessors. However, in this kind of studies, it is almost impossible to blind participants and personnel. Since BMI and BMI *Z* score are objective outcome measures, we assume that lack of blinding of participants, personnel, and assessors is not likely to affect the outcome measures of the intervention. We therefore decided not to downgrade our risk of bias judgement in GRADE based on lack of blinding (Supplementary Table 3(b)).

Our main meta-analyses had generally high statistical heterogeneity, which could be due to variation in control conditions or participant's age range. To minimize heterogeneity, we performed subgroup analyses according to age (mean age < 12 years and ≥12 years). We also performed subgroup analyses according to control conditions ([25], data not shown) and treatment conditions (settings and treatment organization). None of these subgroup analyses were conclusive relative to statistical heterogeneity. As a result of this, we downgraded the quality of the overall documentation in GRADE due to the high statistical heterogeneity in the main outcomes of the meta-analyses.

We used both BMI and BMI *Z* scores as outcomes in our review. BMI may have better sensitivity than BMI *Z* scores to identify changes in children with severe obesity [17]. However, the BMI *Z* scores have the same statistical relation to the distribution of the reference around mean at all ages [94]. This makes results comparable across age groups. In addition, the BMI *Z* scores are sex independent, therefore permitting the evaluation of children's growth status by combining sex and age groups. These characteristics of *Z* scores allow computation of summary statistics at given time points [94]. Our results for BMI and BMI *Z* scores point in the same direction.

One might question the clinical relevance of the decrease in BMI *Z* scores (from −0.12 to −0.16) in our analyses. Others have found that a reduction in BMI *Z* score of −0.25 is necessary to achieve metabolic benefits in adolescents who are obese [95]. However, a Norwegian study found that a reduction in BMI *Z* score of ≥0.1 is sufficient to achieve improvement in cardiovascular risk factors [96]. It is also noteworthy that multicomponent interventions may have other benefits, such as change in physical and sedentary activities and self-esteem, regardless of the change in BMI *Z* scores [97].

All subgroup results should be interpreted with caution due to the heterogeneous nature of the interventions and comparators and the limited number of studies examining some of the subgroups. Additional studies are needed to resolve these questions.

Despite the limitations discussed above, multicomponent lifestyle interventions are important for lifelong habit changes and have fewer complications compared with medication and surgical treatments for overweight and obesity in children and adolescents [98–101]. Therefore, continued efforts are needed to design and implement multicomponent lifestyle interventions for children and adolescents.

## 6. Conclusions

Although the effect is limited, a variety of multicomponent lifestyle interventions involving strategies for change in diet and/or physical activity and family involvement may improve BMI and BMI *Z* score in children and adolescents with varying degrees of overweight and obesity. This positive effect seems to remain at 6-, 12-, and 24-month follow-up compared with standard, minimal, and no treatment. The positive effect on BMI reduction at 6 months seems to be increased when the intervention is given in specialist health care and with a group treatment component included in the intervention. Further efforts to optimize the outcomes of multicomponent interventions are required.

## Supplementary Material



## Figures and Tables

**Table 1:** Overall and subgroup (<12 and ≥12 years) analyses of BMI and BMI *Z* score differences.

Outcome, follow-up	Number of studies	Number of participants	Meta-analyses, mean difference (95% CI)	Statistical heterogeneity		Test for differences between subgroups		GRADE quality of overall documentation
*Overall* Subgroups				*P* value	*I* ^2^ value	*P* value	*I* ^2^ value	
*BMI 6 months*	14	1591	−0.99 (−1.36 to −0.61)	0.00001	72%	—	—	Moderate^1^
Age < 12 years	6	896	−0.74 (−0.26 to −0.22)	0.0006	77%	0.19	40.8%	
Age ≥ 12 years	8	695	−1.26 (−1.85 to −0.68)	0.001	70%			
*BMI 12 months*	19	3238	−0.67 (−1.01 to −0.32)	<0.00001	80%	—	—	Moderate^1^
Age < 12 years	10	2135	−0.31 (−0.45 to −0.17)	0.47	0%	0.13	55.7%	
Age ≥ 12 years	9	1103	−1.02 (−1.95 to −0.10)	<0.00001	87%			
*BMI 24 months*	8	828	−0.96 (−1.63 to −0.29)	0.005	65%	—	—	Low^1,2^
Age < 12 years	3	254	−0.28 (−0.85 to 0.30)	0.26	25%	0.007	86.3%	
Age ≥ 12 years	5	574	−1.58 (−2.33 to −0.83)	0.18	36%			
*BMI Z score 6 months*	18	1607	−0.12 (−0.17 to −0.06)	<0.00001	77%	—	—	Moderate^1^
Age < 12 years	9	696	−0.16 (−0.27 to −0.05)	<0.00001	79%	0.35	0%	
Age ≥ 12 years	9	911	−0.10 (−0.16 to −0.03)	<0.0001	77%			
*BMI Z score 12 months*	22	2804	−0.16 (−0.21 to −0.10)	<0.00001	69%	—	—	Moderate^1^
Age < 12 years	11	1504	−0.17 (−0.26 to −0.07)	0.0006	68%	0.91	0%	
Age ≥ 12 years	11	1300	−0.16 (−0.23 to −0.09)	0.0002	71%			
*BMI Z score 24 months*	8	831	−0.16 (−0.21 to −0.10)	0.17	33%	—	—	High
Age < 12 years	3	257	−0.13 (−0.26 to 0.00)	0.09	58%	0.56	0%	
Age ≥ 12 years	5	574	−0.17 (−0.23 to −0.11)	0.28	21%			

^1^High statistical heterogeneity, ^2^wide confidence interval.

**Table 2:** Subgroup analyses on change in BMI according to setting and treatment organization.

Subgroups	Number of studies	Number of participants	Meta-analyses, mean difference (95% CI)	Statistical heterogeneity		Test for differences between subgroups	
				*P* value	*I* ^2^ value	*P* value	*I* ^2^ value
Change from baseline to 6 months							
*Subgroups on treatment settings*							
Specialist health care	8	816	−1.28 (−1.82 to −0.74)	<0.00001	76%	0.02	68.7%
Primary health care	2	505	−0.10 (−0.64 to 0.43)	0.70	0%		
Schools	3	213	−0.90 (−1.66 to −0.13)	0.02	65%		
Internet	1	57	−0.84 (−1.49 to −0.19)	0.01	NA		
*Subgroups on treatment organization*							
Group treatment	8	827	−1.20 (−1.69 to −0.70)	<0.00001	74%	0.0002	88.4%
Individual treatment	5	698	−0.39 (−0.75 to −0.03)	0.03	0%		
Group and Individual treatment	1	66	−1.61 (−2.09 to −1.13)	<0.00001	NA		

Change from baseline to 12 months							
*Subgroups on treatment settings*							
Specialist health care	6	871	−1.07 (−2.12 to −0.02)	0.05	89%	0.05	62.7%
Primary health care	6	1424	−0.25 (−0.44 to −0.07)	0.007	3%		
Schools	6	886	−0.66 (−1.40 to 0.08)	0.08	81%		
Internet	1	57	−1.36 (−2.28 to −0.44)	0.004	NA		
*Subgroups on treatment organization*							
Group treatment	13	1893	−0.84 (−1.41 to −0.27)	0.004	84%	0.26	26.4%
Individual treatment	5	1254	−0.32 (−0.57 to −0.06)	0.02	32%		
Group and Individual treatment	1	91	−0.41 (−1.18 to 0.36)	0.3	NA		

Change from baseline to 24 months							
*Subgroups on treatment settings*							
Specialist health care	5	546	−0.74 (−1.66 to 0.19)	0.12	75%	0.41	0%
Primary health care	1	151	−1.00 (−2.45 to 0.45)	0.18	NA		
Schools	2	131	−1.59 (−2.45 to −0.72)	0.0003	0%		
*Subgroups on treatment organization*							
Group treatment	7	737	−1.02 (−1.84 to −0.19)	0.02	70%	0.62	0%
Individual treatment	0	0	Not estimable	NA	NA		
Group and Individual treatment	1	91	−0.73 (−1.51 to 0.05)	0.07	NA		

NA = not applicable.

## References

[1] Ng M., Fleming T., Robinson M. (2014). Global, regional, and national prevalence of overweight and obesity in children and adults during 1980–2013: a systematic analysis for the Global Burden of Disease Study 2013. *Lancet*.

[2] Júlíusson P. B., Eide G. E., Roelants M., Waaler P. E., Hauspie R., Bjerknes R. (2010). Overweight and obesity in Norwegian children: prevalence and socio-demographic risk factors. *Acta Paediatrica*.

[3] Centers for Disease Control and Prevention Defining childhood obesity. http://www.cdc.gov/obesity/childhood/defining.html.

[4] Young-Hyman D., Schlundt D. G., Herman L., De Luca F., Counts D. (2001). Evaluation of the insulin resistance syndrome in 5- to 10-year-old overweight/obese African-American children. *Diabetes Care*.

[5] Young T. K., Dean H. J., Flett B., Wood-Steiman P. (2000). Childhood obesity in a population at high risk for type 2 diabetes. *Journal of Pediatrics*.

[6] The N. S., Suchindran C., North K. E., Popkin B. M., Gordon-Larsen P. (2010). Association of adolescent obesity with risk of severe obesity in adulthood. *JAMA*.

[7] Engeland A., Bjørge T., Søgaard A. J., Tverdal A. (2003). Body mass index in adolescence in relation to total mortality: 32-year follow-up of 227,000 Norwegian boys and girls. *American Journal of Epidemiology*.

[8] Must A., Strauss R. S. (1999). Risks and consequences of childhood and adolescent obesity. *International Journal of Obesity and Related Metabolic Disorders*.

[9] Ebbeling C. B., Pawlak D. B., Ludwig D. S. (2002). Childhood obesity: public-health crisis, common sense cure. *Lancet*.

[10] Steinsbekk S., Belsky D., Guzey I. C., Wardle J., Wichstrøm L. (2016). Polygenic risk, appetite traits, and weight gain in middle childhood: a longitudinal study. *JAMA Pediatrics*.

[11] Locke A. E., Kahali B., Berndt S. I. (2015). Genetic studies of body mass index yield new insights for obesity biology. *Nature*.

[12] Whitlock E. P., O’Connor E. A., Williams S. B., Beil T. L., Lutz K. W. (2008). Effectiveness of weight management programs in children and adolescents. *Evidence Report/Technology Assessments No 170 (Prepared by the Oregon Evidence-based Practice Center under Contract No 290-02-0024) AHRQ Publication No 08-E014*.

[13] Ho M., Garnett S. P., Baur L. (2012). Effectiveness of lifestyle interventions in child obesity: systematic review with meta-analysis. *Pediatrics*.

[14] BMJ Best Practice Obesity in children. http://bestpractice.bmj.com/best-practice/monograph/1085/treatment/guidelines.html.

[15] Dinsdale H., Ridler C., Ells L. J. A simple guide to classifying body mass index in children. http://www.thehealthwell.info/node/894424.

[16] Mei Z., Grummer-Strawn L. M., Pietrobelli A., Goulding A., Goran M. I., Dietz W. H. (2002). Validity of body mass index compared with other body-composition screening indexes for the assessment of body fatness in children and adolescents. *American Journal of Clinical Nutrition*.

[17] Kelly A. S., Daniels S. R. (2017). Rethinking the use of body mass index *z*-score in children and adolescents with severe obesity: time to kick it to the curb?. *Journal of Pediatrics*.

[18] Cole T. J., Faith M. S., Pietrobelli A., Heo M. (2005). What is the best measure of adiposity change in growing children: BMI, BMI %, BMI *z*-score or BMI centile?. *European Journal of Clinical Nutrition*.

[19] Dietz W. H., Robinson T. N. (2005). Clinical practice. Overweight children and adolescents. *New England Journal of Medicine*.

[20] Colquitt J. L., Loveman E., O’Malley C. (2016). Diet, physical activity, and behavioural interventions for the treatment of overweight or obesity in preschool children up to the age of 6 years. *Cochrane Database of Systematic Reviews*.

[21] Mead E., Brown T., Rees K. (2017). Diet, physical activity and behavioural interventions for the treatment of overweight or obese children from the age of 6 to 11 years. *Cochrane Database of Systematic Reviews*.

[22] Al-Khudairy L., Loveman E., Colquitt J. L. (2017). Diet, physical activity and behavioural interventions for the treatment of overweight or obese adolescents aged 12 to 17 years. *Cochrane Database of Systematic Reviews*.

[23] Liberati A., Altman D. G., Tetzlaff J. (2009). The PRISMA statement for reporting systematic reviews and meta-analyses of studies that evaluate health care interventions: explanation and elaboration. *Journal of Clinical Epidemiology*.

[24] Oude L. H., Baur L., Jansen H. (2009). Interventions for treating obesity in children. *Cochrane Database of Systematic Reviews*.

[25] Elvsaas I. K., Juvet L. K., Giske L., Fure B. (2016). Effekt av tiltak for barn og unge med overvekt eller fedme [Effectiveness of interventions for overweight or obesity in children and adolescents]. *Rapport*.

[26] Higgins J., Green S. *Cochrane Handbook for Systematic Reviews of Interventions*.

[27] GRADEpro GDT GRADEpro Guideline Development Tool. https://gradepro.org/.

[28] Backlund C., Sundelin G., Larsson C. (2011). Effect of a 1-year lifestyle intervention on physical activity in overweight and obese children. *Advances in Physiotherapy*.

[29] Backlund C., Sundelin G., Larsson C. (2011). Effects of a 2-year lifestyle intervention on physical activity in overweight and obese children. *Advances in Physiotherapy*.

[30] Waling M., Lind T., Hernell O., Larsson C. (2010). A one-year intervention has modest effects on energy and macronutrient intakes of overweight and obese Swedish children. *Journal of Nutrition*.

[31] Waling M., Backlund C., Lind T., Larsson C. (2012). Effects on metabolic health after a 1-year-lifestyle intervention in overweight and obese children: a randomized controlled trial. *Journal of Nutrition and Metabolism*.

[32] Boodai S. A., McColl J. H., Reilly J. J. (2014). National Adolescent Treatment Trial for Obesity in Kuwait (NATTO): project design and results of a randomised controlled trial of a good practice approach to treatment of adolescent obesity in Kuwait. *Trials*.

[33] Croker H., Viner R. M., Nicholls D. (2012). Family-based behavioural treatment of childhood obesity in a UK National Health Service setting: randomized controlled trial. *International Journal of Obesity*.

[34] Davoli A. M., Broccoli S., Bonvicini L. (2013). Pediatrician-led motivational interviewing to treat overweight children: an RCT. *Pediatrics*.

[35] DeBar L. L., Stevens V. J., Perrin N. (2012). A primary care-based, multicomponent lifestyle intervention for overweight adolescent females. *Pediatrics*.

[36] Diaz R. G., Esparza-Romero J., Moya-Camarena S. Y., Robles-Sardin A. E., Valencia M. E. (2010). Lifestyle intervention in primary care settings improves obesity parameters among Mexican youth. *Journal of the American Dietetic Association*.

[37] Ford A. L., Bergh C., Sodersten P. (2010). Treatment of childhood obesity by retraining eating behaviour: randomised controlled trial. *BMJ*.

[38] Gong L., Yuan F., Teng J. (2014). Weight loss, inflammatory markers, and improvements of iron status in overweight and obese children. *Journal of Pediatrics*.

[39] Gourlan M., Sarrazin P., Trouilloud D. (2013). Motivational interviewing as a way to promote physical activity in obese adolescents: a randomised-controlled trial using self-determination theory as an explanatory framework. *Psychology and Health*.

[40] Grey M., Berry D., Davidson M., Galasso P., Gustafson E., Melkus G. (2004). Preliminary testing of a program to prevent type 2 diabetes among high-risk youth. *Journal of School Health*.

[41] Grey M., Jaser S. S., Holl M. G., Jefferson V., Dziura J., Northrup V. (2009). A multifaceted school-based intervention to reduce risk for type 2 diabetes in at-risk youth. *Preventive Medicine*.

[42] Hofsteenge G. H., Chinapaw M. J. M., Delemarre-van de Waal H. A., Weijs P. J. M. (2014). Long-term effect of the Go4it group treatment for obese adolescents: a randomised controlled trial. *Clinical Nutrition*.

[43] Hughes A. R., Stewart L., Chapple J. (2008). Randomized, controlled trial of a best-practice individualized behavioral program for treatment of childhood overweight: Scottish Childhood Overweight Treatment Trial (SCOTT). *Pediatrics*.

[44] Jelalian E., Lloyd-Richardson E. E., Mehlenbeck R. S. (2010). Behavioral weight control treatment with supervised exercise or peer-enhanced adventure for overweight adolescents. *Journal of Pediatrics*.

[45] Lloyd-Richardson E. E., Jelalian E., Sato A. F., Hart C. N., Mehlenbeck R., Wing R. R. (2012). Two-year follow-up of an adolescent behavioral weight control intervention. *Pediatrics*.

[46] Johnston C. A., Tyler C., Fullerton G. (2007). Results of an intensive school-based weight loss program with overweight Mexican American children. *International Journal of Pediatric Obesity*.

[47] Johnston C. A., Moreno J. P., Gallagher M. R. (2013). Achieving long-term weight maintenance in Mexican-American adolescents with a school-based intervention. *Journal of Adolescent Health*.

[48] Johnston C. A., Tyler C., McFarlin B. K. (2007). Weight loss in overweight Mexican American children: a randomized, controlled trial. *Pediatrics*.

[49] Johnston C. A., Tyler C., McFarlin B. K. (2010). Effects of a school-based weight maintenance program for Mexican-American children: results at 2 years. *Obesity*.

[50] Kalarchian M. A., Levine M. D., Arslanian S. A. (2009). Family-based treatment of severe pediatric obesity: randomized, controlled trial. *Pediatrics*.

[51] Kalavainen M. P., Korppi M. O., Nuutinen O. M. (2007). Clinical efficacy of group-based treatment for childhood obesity compared with routinely given individual counseling. *International Journal of Obesity*.

[52] Kalavainen M., Korppi M., Nuutinen O. (2011). Long-term efficacy of group-based treatment for childhood obesity compared with routinely given individual counselling. *International Journal of Obesity*.

[53] Kalavainen M., Utriainen P., Vanninen E., Korppi M., Nuutinen O. (2012). Impact of childhood obesity treatment on body composition and metabolic profile. *World Journal of Pediatrics*.

[54] Kokkvoll A., Grimsgaard S., Ødegaard R., Flægstad T., Njølstad I. (2014). Single versus multiple-family intervention in childhood overweight—Finnmark Activity School: a randomised trial. *Archives of Disease in Childhood*.

[55] Kokkvoll A., Grimsgaard S., Steinsbekk S., Flægstad T., Njølstad I. (2014). Health in overweight children: 2-year follow-up of Finnmark Activity School—a randomised trial. *Archives of Disease in Childhood*.

[56] Looney S. M., Raynor H. A. (2014). Examining the effect of three low-intensity pediatric obesity interventions: a pilot randomized controlled trial. *Clinical Pediatrics*.

[57] Maddison R., Marsh S., Foley L. (2014). Screen-time Weight-loss Intervention Targeting Children at Home (SWITCH): a randomized controlled trial. *International Journal of Behavioral Nutrition and Physical Activity*.

[58] Nguyen B., Shrewsbury V. A., O’Connor J. (2012). Twelve-month outcomes of the Loozit randomized controlled trial: a community-based healthy lifestyle program for overweight and obese adolescents. *Archives of Pediatrics and Adolescent Medicine*.

[59] Nguyen B., Shrewsbury V. A., O’Connor J. (2013). Two-year outcomes of an adjunctive telephone coaching and electronic contact intervention for adolescent weight-loss maintenance: the Loozit randomized controlled trial. *International Journal of Obesity*.

[60] Pakpour A. H., Gellert P., Dombrowski S. U., Fridlund B. (2015). Motivational interviewing with parents for obesity: an RCT. *Pediatrics*.

[61] Patrick K., Norman G. J., Davila E. P. (2013). Outcomes of a 12-month technology-based intervention to promote weight loss in adolescents at risk for type 2 diabetes. *Journal of Diabetes Science and Technology*.

[62] Pbert L., Druker S., Gapinski M. A. (2013). A school nurse-delivered intervention for overweight and obese adolescents. *Journal of School Health*.

[63] Quattrin T., Roemmich J. N., Paluch R., Yu J., Epstein L. H., Ecker M. A. (2012). Efficacy of family-based weight control program for preschool children in primary care. *Pediatrics*.

[64] Quattrin T., Roemmich J. N., Paluch R., Yu J., Epstein L. H., Ecker M. A. (2014). Treatment outcomes of overweight children and parents in the medical home. *Pediatrics*.

[65] Reinehr T., Schaefer A., Winkel K., Finne E., Toschke A. M., Kolip P. (2010). An effective lifestyle intervention in overweight children: findings from a randomized controlled trial on “Obeldicks light”. *Clinical Nutrition*.

[66] Savoye M., Shaw M., Dziura J. (2007). Effects of a weight management program on body composition and metabolic parameters in overweight children: a randomized controlled trial. *JAMA*.

[67] Savoye M., Nowicka P., Shaw M. (2011). Long-term results of an obesity program in an ethnically diverse pediatric population. *Pediatrics*.

[68] Savoye M., Caprio S., Dziura J. (2014). Reversal of early abnormalities in glucose metabolism in obese youth: results of an intensive lifestyle randomized controlled trial. *Diabetes Care*.

[69] Stark L. J., Spear S., Boles R. (2011). A pilot randomized controlled trial of a clinic and home-based behavioral intervention to decrease obesity in preschoolers. *Obesity*.

[70] Stark L. J., Clifford L. M., Towner E. K. (2014). A pilot randomized controlled trial of a behavioral family-based intervention with and without home visits to decrease obesity in preschoolers. *Journal of Pediatric Psychology*.

[71] Steele R. G., Aylward B. S., Jensen C. D., Cushing C. C., Davis A. M., Bovaird J. A. (2012). Comparison of a family-based group intervention for youths with obesity to a brief individual family intervention: a practical clinical trial of positively fit. *Journal of Pediatric Psychology*.

[72] Taveras E. M., Gortmaker S. L., Hohman K. H. (2011). Randomized controlled trial to improve primary care to prevent and manage childhood obesity the high five for kids study. *Archives of Pediatrics and Adolescent Medicine*.

[73] Van Der Baan-Slootweg O., Benninga M. A., Beelen A. (2014). Inpatient treatment of children and adolescents with severe obesity in the Netherlands: a randomized clinical trial. *JAMA Pediatrics*.

[74] Vos R. C., Wit J. M., Pijl H., Houdijk E. C. (2011). Long-term effect of lifestyle intervention on adiposity, metabolic parameters, inflammation and physical fitness in obese children: a randomized controlled trial. *Nutrition and Diabetes*.

[75] Vos R. C., Huisman S. D., Houdijk E. C., Pijl H., Wit J. M. (2012). The effect of family-based multidisciplinary cognitive behavioral treatment on health-related quality of life in childhood obesity. *Quality of Life Research*.

[76] Wake M., Baur L. A., Gerner B. (2009). Outcomes and costs of primary care surveillance and intervention for overweight or obese children: the LEAP 2 randomised controlled trial. *BMJ*.

[77] Wake M., Lycett K., Clifford S. A. (2013). Shared care obesity management in 3-10 year old children: 12 month outcomes of HopSCOTCH randomised trial. *BMJ*.

[78] Williamson D. A., Martin P. D., White M. A. (2005). Efficacy of an internet-based behavioral weight loss program for overweight adolescent African-American girls. *Eating and Weight Disorders*.

[79] Wright K., Suro Z. (2014). Using community-academic partnerships and a comprehensive school-based program to decrease health disparities in activity in school-aged children. *Journal of Prevention and Intervention in the Community*.

[80] Mühlig Y., Wabitsch M., Moss A., Hebebrand J. (2014). Weight loss in children and adolescents. *Deutsches Ärzteblatt International*.

[81] Peirson L., Fitzpatrick-Lewis D., Morrison K., Warren R., Usman Ali M., Raina P. (2015). Treatment of overweight and obesity in children and youth: a systematic review and meta-analysis. *CMAJ Open*.

[82] Snethen J. A., Broome M. E., Treisman P., Castro E., Kelber S. T. (2016). Effective weight loss for children: a meta-analysis of intervention studies 2002–2015. *Worldviews on Evidence-Based Nursing*.

[83] Hayes S., Wolf C., Labbé S., Peterson E., Murray S. (2017). Primary health care providers’ roles and responsibilities: a qualitative exploration of ‘who does what’ in the treatment and management of persons affected by obesity. *Journal of Communication in Healthcare*.

[84] Banks J., Sharp D. J., Hunt L. P., Shield J. P. H. (2012). Evaluating the transferability of a hospital-based childhood obesity clinic to primary care: a randomised controlled trial. *British Journal of General Practice*.

[85] Ickes M. J., McMullen J., Haider T., Sharma M. (2014). Global school-based childhood obesity interventions: a review. *International Journal of Environmental Research and Public Health*.

[86] Kothandan S. K. (2014). School based interventions versus family based interventions in the treatment of childhood obesity: a systematic review. *Archives of Public Health*.

[87] Jelalian E., Rancourt D., Sato A. F. (2013). Innovative interventions in pediatric obesity: commentary and future directions. *Journal of Pediatric Psychology*.

[88] Pedrosa C., Oliveira B. M., Albuquerque I., Simões-Pereira C., Vaz-de-Almeida M. D., Correia F. (2011). Markers of metabolic syndrome in obese children before and after 1-year lifestyle intervention program. *European Journal of Nutrition*.

[89] Pedrosa C., Oliveira B. M., Albuquerque I., Simões-Pereira C., Vaz-de-Almeida M. D., Correia F. (2011). Metabolic syndrome, adipokines and ghrelin in overweight and obese schoolchildren: results of a 1-year lifestyle intervention programme. *European Journal of Pediatrics*.

[90] Kelleher E., Davoren M. P., Harrington J. M., Shiely F., Perry I. J., McHugh S. M. (2017). Barriers and facilitators to initial and continued attendance at community-based lifestyle programmes among families of overweight and obese children: a systematic review. *Obesity Reviews*.

[91] Skelton J. A., Goff D. C., Ip E., Beech B. M. (2011). Attrition in a multidisciplinary pediatric weight management clinic. *Childhood Obesity*.

[92] Staiano A. E., Marker A. M., Comeaux J., Frelier J. M., Hsia D. S., Broyles S. T. (2017). Family-based behavioral treatment for childhood obesity: caretaker-reported barriers and facilitators. *Ochsner Journal*.

[93] Theim K. R., Sinton M. M., Goldschmidt A. B. (2013). Adherence to behavioral targets and treatment attendance during a pediatric weight control trial. *Obesity*.

[94] World Health Organization Global Database on Child Growth and Malnutrition: description: the *z*-score or standard deviation classification system. http://www.who.int/nutgrowthdb/about/introduction/en/index4.html.

[95] Ford A. L., Hunt L. P., Cooper A., Shield J. P. (2010). What reduction in BMI SDS is required in obese adolescents to improve body composition and cardiometabolic health?. *Archives of Disease in Childhood*.

[96] Kolsgaard M. L., Joner G., Brunborg C., Anderssen S. A., Tonstad S., Andersen L. F. (2011). Reduction in BMI *z*-score and improvement in cardiometabolic risk factors in obese children and adolescents. The Oslo Adiposity Intervention Study: a hospital/public health nurse combined treatment. *BMC Pediatrics*.

[97] Kolotourou M., Radley D., Chadwick P. (2013). Is BMI alone a sufficient outcome to evaluate interventions for child obesity?. *Childhood Obesity*.

[98] McDonagh M. S., Selph S., Ozpinar A., Foley C. (2014). Systematic review of the benefits and risks of metformin in treating obesity in children aged 18 years and younger. *JAMA Pediatrics*.

[99] Mead E., Atkinson G., Richter B. (2016). Drug interventions for the treatment of obesity in children and adolescents. *Cochrane Database of Systematic Reviews*.

[100] Ells L. J., Mead E., Atkinson G. (2015). Surgery for the treatment of obesity in children and adolescents. *Cochrane Database of Systematic Reviews*.

[101] Rajjo T., Mohammed K., Alsawas M. (2017). Treatment of pediatric obesity: an umbrella systematic review. *Journal of Clinical Endocrinology and Metabolism*.

