# Supplementary material for: Multicomponent Lifestyle Interventions for Treating Overweight and Obesity in Children and Adolescents: A Systematic Review and Meta-Analyses
Source: J Obes. 2017 Dec 17;2017:5021902. doi: 10.1155/2017/5021902 (PMC5748119; doi:10.1155/2017/5021902)
Supplement: Supplementary file 1 [file 5021902.f1.docx]

## Supplementary Tables

Supplementary Table 1. Description of included studies

| Reference | Number, years and overweight/ obestiy | Intervention | | | | | Comparison | Intervention lenght | Drop out and loss to follow-up | Definition (from included studies):  BMI Z score  ITT analysis |
| --- | --- | --- | --- | --- | --- | --- | --- | --- | --- | --- |
| Backlund 2011a,b, Waling 2011, 2012 Sweden | N=105, 8-12 years  Includes overweight and obesity (age and sex adjusted BMI ≥25) | Family-based multifactor lifestyle intervention, based on principles of behaviour, group treatment and parental involvment. Aimed at achieving lifestyle changes tegarding food habits and physical activity. 14 group meetings. | | | | | Control group with a single information only session before start; “Children allocated to the control group participated in one information session. Apart from that, they only participated in the same measurements as the intervention group.” | 2 year intervention | Drop-out: 0-12 mo: 48%  Drop-out: 0-24 mo: 54%  Reasons for drop-out: not spesified | “The main outcome of the study, BMI, was calculated as weight (kg)/height (m^2^) and converted to BMI *z*-scores by using both US reference data and a Swedish reference dataset.”  “An intention-to-treat analysis with the principle of carrying the last observation forward was performed for children who dropped out before the 1-y follow-up...” |
| Boodai 2014  Kuwait | N=82, 10-14 years  Obesity (≥95 percentile) | Intervention (adapted from the Scottish Childhood Obesity Treatment Trial (SCOTT)). Focus on changing behaviour (reduction of sedentary behaviour, diet and promotion of physical activity). Group sessions (6 sessions, 1 h per session) for adelescents (boys and girls separate) and parents. | | | | | Control group  Referral to primary care in Kuwait; “Adolescents, and their parents, who were allocated randomly to the control group were therefore informed that they were obese and advised to attend primary care.” | 6 mo intervention | Drop-out 0-6 mo: 23%  Reasons for drop-out: unable to contact | “BMI Z scores were calculated based on US CDC 2000 reference  Data.”  “The analysis was intention-to-treat, where we used data from all adolescents for whom data were available on the basis of the group to which they were allocated, regardless of their adherence to the protocol (attendance).” |
| Croker 2012 United Kingdom | N=72, 8-12 years  Overweight and obesity according to International Obesity Task Force | Family-based behavioural Treatment, a structured intervention comprising advice on whole-family lifestyle change with a behavioural weight control programme for the overweight child. Based on learning theory and uses behaviour modification techniques. 15 sessions á 1½ h (10 weekly, 3 fortnightly, 2 monthly). | | | | | Waiting list control. | 6 mo intervention | Drop-out 0-6 mo: 22%  Reasons for drop-out: dropped out before start, health problems for mother, time problems, did not want to continue, new baby in the family, unknown reason | “Standard deviation scores for BMI, weight, height, and waist were calculated from raw values by adjusting for age and gender using British 1990 reference data. The LMSgrowth macro was used.”  “All 6 month outcomes were analysed on an ITT basis using baseline values carried forward if outcome data were missing.” |
| *Davoli 2013  Italy | N=372, 4-7 years  Overweight (≥85 to 95 percentile) | Family-based lifestyle intervention including motivational interview, and goal setting. 5 individual meetings based on the transtheoretical model of addiction and behavior change. The child and parents always had to leave the meeting having agreed on 2 objectives (1 concerning food and 1 concerning PA improvements) that were clearly defined and achievable. | | | | | Control group received standard care currently offered by pediatricians to overweight children and a booklet with the main information on obesity prevention. | 12 mo intervention | Drop-out: 0-12 mo: 4,6%  Reasons for drop-out: change of doctor, moved, did not want to continue, illness, problems in collecting data | “BMI z scores and changes from overweight status to normal weight or obesity were also reported to allow comparability with previous studies.“  “To perform an intention-to-treat analysis for the primary objective, missing values for ∆BMI were replaced with the mean variation of BMI calculated in the control group.” |
| DeBar 2012 USA | N=208, 12-17 years  Obesity (≥90 percentile)  Intervention aimed at girls (and their family) | Lifestyle intervention in primary care, 16 group sessions (á 90 min) for the girls (weekly for the first 3 months, bi-weekly thereafter), 12 sessions for parents. Intervention included: change in dietary intake and eating patterns; increasing physical activity; addressing issues associated with obesity in adolescent girls (eg, depression, disordered eating patterns, poor body image); and training participants’ PCPs to support behavioral weight management goals collaboratively. | | | | | Usual care control group; written material (incl outlines of evidence-based appraches to weight management, a parents guide to help adolescents make healthy lifestyle changes, local resources for weight management and healthy activity, and suggested books and online materials on healthy lifestyle changes), 1 meeting with primary care provider. | 5 mo intervention, follow-up at 6 and 12 mo | Drop-out: 0-6 mo: 6%  Drop-out: 0-12 mo: 17%  Reasons for drop-out: not specified | “BMI was converted to z score during analysis.”  “….all randomized participants were included in the analyses consistent with an intention-to-treat approach” |
| *Diaz 2010 Mexico | N=76, 9-17 years  Obesity (≥95 percentile) and obesity (≥90 percentile) + waist circumference (≥90 percentile) | Lifestyle intervention, 12 group sessions (á 2 h) of behavioural curriculum, dietary advice from a dieterian and monthly consultation with primary care physician. The behavioural componenet was based on the workbook “Programa Cambia”, adapted from Mellin’s “Shapedown program”. | | | | | Participants and their parents attended monthly consultation (10-15 min) with a primary care physician that encouraged youths to performe 30 min of physical activity most days of the week, limit sedentary time to 2 hours per day, and follow a diet consistent with the Food Guide Pyramid. Also parents were encouraged to adopt positive behaviours. | 12 mo intervention | (Drop-out 0-6 mo: 34%)  Drop-out 0-12 mo: 43%  Reasons for drop-out: not specified | “BMI and BMI z score were obtained using Epi Info software.”  “We also applied an intention-to-treat analysis at 12 months in the primary outcomes of the study. Considering the risk of bias of procedures for analyzing incomplete data [..], we made an effort to obtain the primary outcomes [..] of all participants who dropped out of the study (n=33) measureing children at their homes.” Leading to 87% included in the ITT-analysis. |
| *Ford 2010 United Kingdom | N=106, 9-17 years  Obesity (≥95 percentile) | Mandometer training to slow down speed of the eating and reduce food intake + family-based lifestyle intervention; 4 meetings including motivational interview. Motivational interviewing were used to engage participants and families in the decision making process for lifestyle changes. | | | | | Family-based lifestyle intervention; 4 meetings including motivational interview. Motivational interviewing were used to engage participants and families in the decision making process for lifestyle changes. | 12 mo intervention | Drop-out: 0-12 mo: 14%  Reasons for drop-out: not specified | “BMI was adjusted for age and sex to give a BMI SDS with British 1990 growth reference data from the Child Growth Foundation.”  “…and the second for all participants who attended for follow-up measures, regardless of treatment completion, using the last available data for analysis. As the children who dropped out before 12 months had a slightly higher initial mean BMI SDS than the others (see below), we used analyses of covariance to adjust for baseline values in comparisons between 12 month means. |
| *Gong 2014  China | N=326, 7-11 years  Overweight and obesity (BMI for age Z score >1) | Nutrition-based comprehencive intervention including nutrition education and promotion of healthy eating behaviours, and physical activity (PA) (“Happy 10” campaign” – developed to promote PA in school-aged children). Involvment of parents (3 lectures of nutrition and health). Also education of teachers and kitchen personell. | | | | | No intervention control group. | 12 mo intervention | Drop-out 0-12 mo: no drop-out | Chinese reference population were used for BMI z score.  No drop-outs. |
| Gourlan 2013  France | N=54, 11-18 years  Obesity (≥90 percentile) | Motivational interview (6 phone sessions) as addition to standard weight reduction program (two sessions). The standard weight loss programs goal was to promote a balanced diet, a healthy lifestyle and physical activity. | | | | | Standard weight reduction program (two sessions). The standard weight loss programs goal was to promote a balanced diet, a healthy lifestyle and physical activity. | 6 mo intervention | Drop-out 0-6 mo: 13%  Reasons for drop-out: moved, family problems, own decision | “All primary outcomes analyses are based on an intetntion to treat analysis, with all adolescents included in the condition they were assigned.” |
| Grey 2004 USA | N=41, 10-14 years  Obesity (≥95 percentile)  Inner city youth at high risk for type 2 diabetes mellitus | Preliminary study: School-based intervention  Coping skills training (CST) + nutrition education (a family-cdentered culturally sensitive interactive nutrition curriculum) and physical activity (2d/week á 45 min) + telephone support (once a week). Parental involvment; could participate in nutrition educational program and encouraged to collaborate with their adolescents to increase physical activity and decrease sedentary behaviour. | | | | | Control group with the same nutrition education and physical activity as the intervention group + telephone support (once a month). Also same parenal involvment as intervention group. | 16 weeks afterschool program, follow up at 12 mo | Drop-out: 0-6 mnd: 0%  Drop-out: 0-12 mo: 22%  Reasons for drop-out: chose not to complete data at 12 mo | “BMI was calculated using the American Medical Association chart then rechecked using the formula BMI=kg/m^2^.”  No drop-outs at 6 mo. |
| Grey 2009 USA | N=198, students in seventh grade, mean gj. 12,8 (±0,7) years  Overweight and obesity (from ≥85 percentile)  Inner city youth at high risk for type 2 diabetes mellitus | School-based intervention  Coping skills training (CST) intervention. 8 classes with nutrition (a non-diet, family-centered approach) and activity educational component (focusing on reducing leisure-time sedentary behaviours, and creative ways to increase physical activity) + 5 classes CST and 9 mo weekly telephone health coaching. (4 schools). Parental involvment. | | | | | General education (GE) intervention. 8 classes with nutrition and activity educational component (2 schools), same as intervention group schools. | 16 weeks afterschool program, follow up at 12 mo | Drop-out: 0-4 mnd: 14%  Drop-out: 0-12 mnd: 24%  Reasons for drop-out: not specified | “The intent-to-treat analysis was performed on all 198 randomized subjects. This included 44 children who were randomized and participated in the evaluation, but did not attend the intervention classes.” |
| Hofsteenge 2014  The Netherlands | N=122, 11-18 years  Overweight and obesity (according to Cole et al. [102]) | Intervention (Go4it); 7 sessions (duration 90 min) on healthy dietary, sedentary, and physical activity behaviour + cognitive behavioural therapy in which they learned how to improve their lifestyle and how to maintain energy balance. Four booster group sessions in order to encourage the adolescents to maintain or further improve their energy  Carried out in an outpatiet clinic.  balance behaviour and discuss problems and questions. 2 sessions for parents. | | | | | Regular care in the Netherlands (valid for year 2006-09); referral to a dietitian in the home care setting. | 6 mo intervention (3 mo intensive + 3 mo with booster-sessions), follow-up at 18 mo. | Drop-out: 0-6 mo: 20%  Drop-out: 0-18 mo: 44%  Reasons for drop-out: loss of interest, not reachable, family problems, illness not related to study | “For calculation of BMI standard deviation scores (BMIsds) or z-scores, a reference database of Dutch children was used..”  “Group comparisons were performed according the intention-to-treat principle whereby all subjects were analyzed in the group to which they were randomly assigned.” |
| Hughes 2008 United Kingdom | N=134, 5-11 years  Obesity (≥98 percentile) | «Best practice» Individualized Behavioral Program, a family centered apporach where the child and family took control of own lifestyel changes in diet, physical activity and sedentary behavior. 1-to-1 contact between family and dietitian. 8 appointments. Total patient contact time of ~5 h. | | | | | Standard dietetic care; 3-4 outpatient appointments by pediatric dietitians, total patient contact time of ~1,5 h. Involved a didactic «medical model». | 6 mo intervention, follow-up at 12 mo | Drop-out: 0-6 mo: 28% Drop-out: 0-12 mo: 36%  Reasons for drop-out: failed to attend further appointments , lost to follow-up | “BMI and hight were expressed relatove to United Kingdom reference data as z scores.”  “The primary analysis was performed on an intention-to-treat basis for each outcome measure and involved all participants who attended for follow-up measures, regardless of whether they completed the treatmen” |
| Jelalian 2010 + Lloyd-Richardson 2012 USA | N=118, 13-16 years  Overweight/obesity (between 20% and 80% over mean BMI)  Data from Lloyd-Richardson 2012 | Group based cognitive behavioral treatment combined with peer enhanced adventure therapy, for adolescents and a primary caregivert  Both group-based interventions (intervention and control) included 16 one-hour weekly sessions, with parents and adolescents attending separate concurrent meetings, followed by four bi-weekly maintenance sessions. | | | | | Group based cognitive behavioral weight control treatment combined with supervised aerobic exercise, for adolescents and a primary caregiver | 4 mo intervention. Measurements at 12 and 24 mo | Drop-out: 0-4 mo: 15%  Drop-out: 0-12 mo: 21%  Drop-out: 0-24 mo: 25%  Reasons for drop-out: no longer interested, time conflict, non-study illness, moved out of area, failed to respond to calls | “Height and weight were used to calculate body mass index (BMI; kg/m2), standardized BMI score (z-BMI), as well as percent over BMI.”  “Analyses included all randomized participants (intent to treat; n=118), assuming no change from baseline for noncompleters….” |
| *Johnston 2007a, 2013  USA | N=71, 10-14 years  Overweight and obesity (from ≥85 percentile) | School based intervention  Instructor-led intervention sessions; nutrition classes once a week and a physical training class 4 days a week. 12-weeks of daily sessions (mon to fri) and 12 weeks of bi-weekly sessions. Parents attended monthly meetings. | | | | | Self-help only condition (participants and their parents), a parent-guided manual. Aim: improving diet quality and increasing time spent doing physical activity, 12 weekly sessions followed by maintenance activities. | 6 mo intervention; 12 and 24 mo follow-up | Drop-out: 0-12 mo: 8%  Drop-out: 0-24 mo: 18%  Reasons for drop-out: discontinued intervention, no longer at school | “Body mass index (BMI) was calculated using measured hight and weight, and was standarized (zBMI) using age and gender normative data from the Centers for Disease Control and Prevention.”  “As recommended by the CONSORT guidelines for randomized trials, models were developed for both completers and intention-to-treat (ITT) using the baseline carried forward (BCF) method.” |
| *Johnston 2007b, 2010 USA | N=60, 10-14 years  Overweight and obesity (from ≥85 percentile) | School based intervention  Intensive behavioural weight management program; participants received nutrition instruction (1 day/week) and physical activity training (4 days/week) for 12 weeks, followed by 12 weeks of bi-weekly sessions. Parents attended monthly meetings. | | | | | Self-help condition, a 12-week parent-guided manual intended to promote child weight loss followed by maintenance activities. | 6 mo intervention, follow-up at 12 and 24 mo. | Drop-out: 0-6 mo: 5%  Drop-out: 0-12 mo: 5%  Drop-out: 0-24 mo: 10%  Reasons for drop-out: not specified | “BMI was calculated by using  measured height and weight and was standardized (*z*BMI) by using age- and gender-normative data from the Centers for Disease Control and Prevention “  “As recommended by the Consolidated Standards of Reporting Trials guidelines for randomized trials, models were developed for both completers and intention to treat using the last observation carried forward method.” |
| *Kalarchian 2009 USA | N=192, 8-12 years  Obesity (≥97 percentile) | Family-Based Intervention consisted of 20 group meetings (60 minutes each). Adult and child met separately and were presented with complementary material. They met together with a lifestyle coach to review self-monitoring records and to set weekly goals. Six booster sessions (3 group sessions and 3 telephone calls). | | | | | Usual care condition were offered 2 nutrition consultation sessions to develop an individual nutrition plan based on the Stoplight Eating Plan. There was no additional contact between assessments. | 12 mo intervention, most intensive the first 6 mo | Drop-out: 0-6 mo: 16%  Drop-out: 0-12 mo: 28%  Reasons for drop-out: not specified | “The mixed model is an ITT approach, because the analysis includes all subjects with varying numbers of assessments, with the assumption that the incomplete data are missing at random. To examine the missing-at-random assumption and the stability of results, we performed a series of sensitivity analyses [..] To evaluate the impact of missing data further, we used models in which subject data were weighted by a function of the probability of being missing. Because the overall pattern of results from sensitivity analyses and weighted models was similar to that from the primary ITT analyses, we report the results of the primary analyses only.” |
| *Kalavainen 2007, 2011, 2012 Finland | N=70, 7-9 years  Obesity (weight for height of 120% to 200%) | Family-centered group program: based on the principles of behavioral and solution-oriented therapy. Focused on promoting a healthy lifestyle and well-being of obese children instead of weight management. Consisted of 15 sessions of 90 min in duration held separately for parents and children, except one joint session. | | | | | Routine program was modified from current counseling practice for obese children in school health care in Finland. The program consisted of booklets for families and two individual appointments for each child by school nurses. | 6 mo intervention | Drop-out: 0-6 mo: <3%  Drop-out: 0-24 mo: <3%  Drop-out: 0-36 mo: <3%  Reasons for drop-out: not specified | “..(BMI-SDS), also called Z-score, by using an obesity calculator. The calculator uses the British gender-specific growth reference from 1990, produced by the LMS method, and revised in 1996. The LMS method summarizes the distribution of BMI at each age by its median (M), coefficient of variation (S) and a measure of skewness expressed as a Box–Cox power (L) required to transform the data to normality.“  Primary outcomes were evaluated with an intent-to-treat (ITT) analysis. |
| Kokkvoll 2014a og b  Norway | N=97, 6-12 years  Overweight and obesity (corresponding to BMI 27,5 for adults) | Multiple-family intervention; clinical interview and examination, and outlining of definite aims. 3-day inpatient programme focusing on physical activity and healthy food, group sessions with other families and a multidisciplinary hospital team, municipality follow-up including individual (30 min) and groupwise counselling (1 h), group-based physical activities twice weekly (each 1 h), family participation in a 4-day camp after 4–6 months. | | | | | Single-family intervention; clinical interview and examination, and outlining of definite aims towards the next consultation. Families met with a nutritionist after 1–2 months. Follow up by public health nurse in their own municipality, and at the hospital. | 12 mo intervention | Drop-out: 0-12 mo: 20%  Drop-out: 0-24 mo: 29%  Reasons for drop-out: long journey, family problems | “BMI SDS extracted from an obesity calculator based on British reference data”  "All data were analysed by the intention-to-treat principle.” |
| Looney 2014  USA | N=22, 4-10 years  Overweight and obesity (from ≥85 percentile) | Newsletter + growth monitoring + family based behavioural strategies to change dietary and lesure-time activities. Growth monitoring and family strategies were given in in-person appointments and in phone calls. | | | Newsletter + growth monitoring condition: monitoring and monthly feedback on child’s growth in in-person appointments and in phone calls. | | Newsletter condition  All groups attended standard care program and reseived the same 6 educational newsletters on nutrition and leisure-time activity topics. | 6 mo intervention | Drop-out 0-6 mo: 4,5% (1 of 22)  Reasons for drop-out: not specified | “…the child’s BMI value was standarized in relation to the population mean and standard derivation for the child’s age and sex to determine z-BMI.”  “Intent-to-treat analyses were conducted with baseline values carried forward for participants lost to follow-up.” |
| Maddison 2014  New Zealand | N=251, 9-12 years  Overweight and obesity (according to Cole et al. [102]) | SWITCH (Screen-time weight-loss intervention targeting children at home) – home-based, family-delivered intervention to reduce screen-based sedentary behaviour.  Participants in both groups also received a phone call at 12 weeks to monitor any adverse events. | | | | | No intervention / wait list control.  The control group was asked to continue with their usual behaviour and had access to a generic SWITCH public website. | 6 mo intervention | Drop-out: 0-6 mo: 5%  Reasons for drop-out: not specified | “..BMI z-score (standardized by age and sex using 2007 WHO growth reference),..”  “Treatment evaluations were performed on the principle of intention to treat (ITT), using data collected from all randomised participants. A multiple imputations method was applied to the missing data (if any) for the primary outcome.” |
| Nguyen 2012, 2013 Australia | N=151, 13-16 years  Overweight and obesity (BMI Z scores between 1,0 to 2,5) | Loozit group program and additional therapeutic contact (ATC); 7x75 min weekly group sessions + booster sessions 7x60 min for 22 mo + additional therapeutic contact, a total of 32 SMS/e-mails and 14 phone coaching sessions.  *The Loozit study*: separate meetings for parents/caregivers and adolescents in phase 1. In phase 2, adolescents attended booster group sessions. | | | | | Loozit group program; 7x75 min weekly group sessions + booster sessions 7x60 min for 22 mo | 24 mo intervention (Phase 1: 2 mo intervention. Phase 2: maintenance phase from 2-24 mo) | Drop-out: 0-12 mo: 5%  Drop-out: 0-24 mo: 11%  Reasons for drop-out: could not find  childcare, did not want to participate  any longer, transport difficulties, difficult family situation, post-baseline leg injury, could not be contacted despite repeated attempts | “The BMI *z* scores were calculated based on age- and sex-specific reference values. (CDC growth charts: United  States.)”  “Consistent with an intent-to-treat approach, all available data for participants as originally randomly assigned were retained.” |
| *Pakpour 2015  Iran | N=357, 13-18 years  Obesity (≥95 percentile) | **Interventon 1:**  Motivational interviewing (a client-centered clinical method to faciliate behavioural change) including parental involvment (MI + PI) | | | **Intervention 2:**  Motivational interviewing (MI) only | | No intervention control group | 12 mo intervention | Drop-out 0-12 mo: 3%  Reasons for drop-out: not specified | “A series of intention-to-treat, 2-way repeated measures analysis were performed with intervention [..] “ |
| Patrick 2013  USA | N=101, 12-16 years  Overweight and obesity (from ≥85 percentile)  Adolescents at Risk for Type 2 Diabetes. | **Intervention 1**:  Website program (designed to promote weight loss and healthy behaviors) + monthly 90 min group sessions for adolescents and parents, including nutrition demonstrations and physical activities. Bi-monthly phone calls. | **Interventon 2:**  Website (designed to promote weight loss and healthy behaviors) program + SMS (minimum three per week related to challenges and intervention goals. Reminder SMS if not logging on to the website. Also possible to communicate via SMS with a health counselor.) | | | **Intervention 3:**  Website program (designed to promote weight loss and healthy behaviors) | Usual care:  Printed materials. Participants were encouraged to attend three 1 h group nutrition sessions during the first 6 weeks. They also received monthly tip sheets by mail. | 12 mo intervention | Drop-out 0-12 mo: 37%  Reasons for drop-out: discontinued, lost to follow-up | “National norms from the Centers for Disease Control and Prevention were used to calculate BMI z-scores using age- and sex-specific median, standard deviation..”  "Intent-to-treat analyses were conducted using all available data from participants who enrolled, were randomized, and started the interventions (n = 101) assuming data were missing at random." |
| Pbert 2013  USA | N=82, in 9.-11. grade, ca 15-16 years  Overweight and obesity (from ≥85 percentile) | School intervention, school-nurse delivered  Intervention - “Lookin' Good Feelin' Good”, 6 one-on-one counseling sessions with a patient-centered counseling approach. The intervention was based on Social Cognitive Theory. | | | | | Control group - 6 one-on-one visits to be weighed, review behavior changes, read 6 informational pamphlets on weight management, and have questions answered. | 2 mo intervention, follow-up at 6 mo | Drop-out 0-6 mo: no drop-out | No drop-outs  “BMI was calculated as weight (kg)/height squared (in meters) for age and gender using the CDC BMI charts.” |
| *Quattrin 2012, 2014  USA | n=96, 2-5 years  Overweight and obesity (from ≥85 percentile).  And an overweight parent. | Family-based intervention program. The group leader emphasized parenting and behavioural strategies to promote child and parent behavior change, including parenting-related techniques (selective ignoring, time out, praising, rewarding, contracting) and strategies aimed at changing parental behavior that would facilitate parent and child change.  Both treatment arms included ten 60-minute sessions (4 weekly, 2 bimonthly, and 4 monthly) in which a group leader delivered dietary/physical and sedentary activities education, and trained staff engaged the children in active games. | | | | | Information control | 12 mo intervention, follow-up at 24 mo | Drop-out 0-6 mo: 9%  Drop-out 0-12 mo: 18%  Drop-out 0-24 mo: 28%  Reasons for drop-out: moving out of state, parent had bariatric surgery, parental separation, change in work schedule, felt program to different from what they hoped | “Height and weight were used to calculate BMI, z-BMI, and %0BMI.”  “An intention-to-treat analysis was performed by using mixed analysis of variance models to test changes in child percent over BMI (%0BMI) and z-BMI and to explore potential moderators of group differences in treatment response.” |
| *Reinehr 2010 Germany | N=66, 8-16 years  Overweight/obesity (>90 to < 97 percentile) | «Obeldicks light» based on physical activity, nutrition education, and behaviour counselling. 37 sessions for children, 6 sessions for parents, and 5 sessions for parents and children, total of 67 h for each family. | | | | | No intervention control group, (waiting period of 6 mo). | 6 mo intervention | Drop-out 0-6 mo: 8,5%  Reasons for drop-out: not specified | “The degree of overweight was quantified using Cole’s least mean square method,which normalized the BMI skewed distribution and expressed BMI as a standard deviation score (BMI-SDS). Reference data for German children were used.“  “In order to follow an intention-to-treat analysis approach (ITT) all missing values at follow-up due to drop-out were set back to baseline values...” |
| *Savoye 2007, 2011 USA | N=209, 8-16 years  Obesity (≥95 percentile) | Bright Bodies Weight Management Group. Twice a week for 6 months and then every other week for 6 months. First, the program consisted of exercise twice (50 minutes each) and nutrition/behavior modification once (40 minutes each) per week. Participants and caregivers attended all classes, including nutrition-related topics, together, but behavior modification classes for participants and caregivers were held separately. | | | | | Control group, were seen in the pediatric obesity clinic every 6 months and received diet and exercise counseling by registered dietitians and physicians along with brief psychosocial counseling by a social worker. | 12 mo intervention, follow-up at 24 mo | Drop-out 0-6 mo: 22%  Drop-out 0-12 mo: 32%  Drop-out 0-24 mo: 57%  Reasons for drop-out: lost interest, moved, family issue, transportation, schedule conflict, lost to follow-up | “An intent-to-treat analysis was performed. [..]. Multiple imputation with data augmentation under the multivariate normal model using PROC MI from SAS was performed to impute missing outcome data.” |
| *Savoye 2014  USA | N=75, 10-16 years  Obesity (≥95 percentile)  Obese adolescents with elevated 2-h glucose levels. | Yale’s Bright Bodies (BB) Program, a family-based, lifestyle intervention tailored for inner-city minority children and their families. The program consisted of two 50-min exercise sessions per week, one weekly weigh-in, and a 40-min nutrition/behavior modification class. | | | | | Standard clinical care, diet and exercise instruction by dietitian. Sedentary activities were discouraged, and activities the child enjoyed were encouraged. | 6 mo intervention | Drop-out 0-6 mo: 23%  Reasons for drop-out: transportation issues, broken ankle, psychiatric hospitalization, started prednisolone, started metformin, lost to follow-up. | “BMI z scores were based on the Centers for Disease Control and Prevention growth charts.”  “The primary analysis was conducted based on the intent-to-treat principle, with participants analyzed in their original randomized group. [..]. Given that 6-month follow-up assessments were missing in 17 (23%; 7 BB, 10 CC) individuals, multiple imputation with data augmentation under the multivariate normal model by PROC MI (SAS Institute, Cary, NC) was performed.” |
| *Stark 2011 USA | N=18, 2-5 years  Obesity (≥95 percentile)  And an overweight parent | Learning about activity and understanding nutrition for child health (LAUNCH). Phase 1 (Intensive Intervention) was 12 weekly sessions that alternated between group-based clinic sessions (parent and child concurrent groups) and individual home visits. Phase 2 (Maintenance) was 12 weeks of every other week sessions, alternating between group sessions in clinic and home sessions. | | | | | Standard of care. Pediatricial counseling; designed to deliver dietary and physical activity recommendations. A certified pediatrician met each family individually for one 45-min visit. | 6 mo intervention, follow-up at 12 mo | Drop-out 0-6 mo: 5,5%  Drop-out 0-12 mo: 11%  Reasons for drop-out: job loss, unable to travel to assessment visit, did not come in for weight, did not wear actigraph | “Children’s BMI *z*-score and BMI percentile for sex and age were calculated using the Centers for Disease Control and Prevention growth curves.”  “..using intent-to-treat analysis.[..]. The stability of the results for the primary outcomes to missing data were examined through imputing missing change scores based on the regression of the change scores on the baseline scores.” |
| *Stark 2014  USA | N=42, 2-5 years  Obesity (≥95 percentile)  And an overweight parent | Clinic and Home based behavioural intervention (LAUNCH-HV). Phase 1: 12 weekly sessions (parent and child concurrent groups), and individual home visits. Phase 2: 12 weeks of every-other-week sessions (clinic and home)  LAUNCH = Learning about Activity and Understanding Nutrition for Child Health | | Clinic based intervention (LAUNCH-clinic). Intervention content was identical to LAUNCH-HV). No home visits. Conducted every other week during months 1-3 and monthly during months 4-6. | | | Pediatrician counseling, manualized intervention designed to deliver dietary and physical activity recommendations. A certified pediatrician met each family individually for one 45-min visit. | 6 mo intervention | Drop-out 0-6 mo: 33%  Reasons for drop-out: time/session schedule, work conflicts, unable to contact, family illness, dissatisfied with group allocation, dissatisfied with information received at visit, transportation difficulties, child behaviour problems-beginning therapy, child illness. | “Measurements were averaged to calculate the children’s BMIz and BMI percentile for sex and age using the Centers for Disease Control and Prevention growth curves.”  “..using intent-to-treat (ITT) analyses... [..]. PROC MIXED using maximum likelihood (ML) setimation was used to account for any missing data due to dropout in the analyses under the missing at random (MAR) assumption.” |
| Steele 2012 USA | N=93, 7-17 years  Overweight and obesity (from ≥85 percentile) | Positively fit; family-based behavioural group intervention. 10 weekly group treatment sessions lasting approximately 90 min. Separate sessions for children (7–12) and adolescents (13–17) and parents. Each treatment session consisted of nutrition/physical activity education followed of behavioral intervention, with summary and goal-setting period at the conclusion of each session. | | | | | Brief family intervention;Trim Kids treatment program. Families received three 60-min individual face-to-face visits with  Dietitian. | 10 weeks intervention, follow-up at 12 mo | Drop-out 0-10 weeks: 17%  Drop-out 0-12 mo: 26%  Reasons for drop-out: did not respond to follow-up requests | “Conversion of height, weight, sex, and age values to standardized BMI scores (zBMI) was performed using a SAS application provided by the Centers for Disease Control and Prevention.”  "This study employed an intent-to-treat strategy (ITT) [..].Participant attrition resulted in missing data that were accounted for statistically using Full Information Maximum Likelihood" |
| Taveras 2011 USA | N=475, 2-6 years  Overweight and obesity (from ≥85 percentile)  Included at least one overweight parent | Primary care-based obesity intervention: The high five for kids study. Families received motivational interviewing by clinicians and educational modules targeting television viewing and fast food and sugar-sweetened beverage intake | | | | | Usual care; families received the current standard of care offered by their pediatric practice. This included well-child care visits and follow-up appointments for weight checks with their pediatrician or a nutritionist. | 12 mo intervention | Drop-out 0-12 mo: 6%  Reasons for drop-out: not specified | “We calculated BMI and age- and sex-specific BMI *z* scores and percentiles.”  “In intent-to-treat analyses, we used crude and adjusted multivariate regression models, corrected for clustering by practice, to examine differences from baseline to 1 year between the intervention and usual care groups.” |
| Van Der Baan-Slootweg 2014  Netherlands | N=90, 8-18 years  Obesity (≥98,9 percentile) | Inpatient treatment program (6 months of hospitalization on working days). They followed a program during weekdays and returned home for the weekends with homework assignments. The program consisted of an exercise schedule 4 days per week (30 to 60 min each day with a mean duration of 45 min for each exercise session) and nutrition/behavior modification once per week (60 min for each session). Patients and caregivers received comparable information about nutrition and behavior, but the 1-hour lessons were held separately, at 3 times during the treatment period.  Both treatment programs involved an intensive, family-based, lifestyle intervention, including exercise, nutritional education, and behavior modification for the patients and their caregiver(s). | | | | | Ambulatory treatment program (12 days of hospital visits at increasing intervals during a 6-month period). Patients attended the program for 12 visits at increasing intervals for 6 months. The children exercised for an hour and attended an educational program for 1 h and a nutritional educational session for half an hour. Children and parents were encouraged to exercise at home on 3 additional days per week and to reduce sedentary behaviors. In parallel sessions, caregivers were given detailed instructions on nutrition and nutritional behavior. | 6 mo intervention, follow-up at 12 and 30 mo | Drop-out 0-6 mo: 12%  Drop-out 0-12 mo: 44%  Drop-out 0-30 mo: 34%  Reasons for drop-out: homesickness, motivational problems, behavioural problems, family problems | “The primary outcome of the study was the BMI *z* score, standardized by the use of age- and sex-normative data from the Dutch National Growth Study of 1997 ”  “Data were analyzed using the intention-to-treat principle.” |
| Vos 2011, 2012  Netherlands | N=81, 8-17 years  Obesity (according to Cole et al. [102]) | Family-based cognitive behavioural multidisciplinary lifestyle treatment; a screening phase of individual counselling of the children with their parents, followed by an intensive phase of group sessions (seven group meetings for the children, five separate parent meetings and one parent meeting together with the children). | | | | | Control group; received standard care and advice, at start of the trial, on how to increase their physical activity, decrease their sedentary activities and improve their eating behaviour. | 3 mo intervention, follow-up at 12 mo | Drop-out 0-12 mo: 17%  Reasons for drop-out: not specified | “BMI was expressed as standard deviation score (SDS) for Dutch references.”  “Missing data in the current study were assumed “missing at random”. All available data of the subjects, irrespectively if data were not complete for all occasions, were analyzed according to the treatment protocol they were randomized to.” |
| Wake 2009  Australia | N=258, 5-10 years  Overveight and obesity (up to BMI Z score 3,0) | The LEAP 2 randomised controlled trial  Intervention with a behavioural epidemiology framework; four standard consultations targeting change in nutrition, physical activity, and sedentary behaviour, supported by purpose designed family materials. | | | | | Control group; received control care. | 3 mo intervention, follow-up at 6 og 12 mo | Drop-out 0-6 mo: 3%  Drop-out 0-12 mo: 6%  Reasons for drop-out: declined or missed sessions, uncontactable, overseas | “BMI z score was also calculated using the US Centers for Disease Control (CDC) 2000 sex-specific BMI-for-age growth charts.”  Participants were analysed in the groups to which they were randomised. |
| Wake 2013  Australia | N=118, 3-10 years  Obesity (≥95 percentile) | HopSCOTCH randomised trial  Shared care-model; one tertiary appointment followed by up to 11 general practice consultations, supported by shared care, web based software in were data from each appointment were enterd.  he HopSCOTCH is a web based, shared care software designed to provide collaboration and communication between the specialists and general practitioners. | | | | | Usual care, participants were free to seek assistance from their general practitioner or from any other service | 12 mo intervention | Drop-out 0-12 mo: 9%  Reasons for drop-out: withdrew, not contactable | “…from which the researchers calculated body mass index z scores and body mass index status according to the US Centers for Disease Control reference.”  “We analysed data by using the intention to treat principle…” |
| *Williamson 2005 USA | N=57, 11-15 years  Overweight and obesity (from ≥85 percentile)  Only girls included. Parents were also participants in the study. | Interactive behavioural internet program with internet counselling. Participants met in face-to-face sessions on four accoasions. The intervention was based on the family treatment methods by Epstein et.al. | | | | | Control condition; an internet (non-interactive) health education program. Participants met in face-to-face sessions on four accoasions. | 6 mo intervention | Drop-out 0-6 mo: 12%  Reasons for drop-out: not specified | "An intention-to-treat analysis was used for all data analysis, i.e., at 6 months, the baseline value was carried forward to the 6-month assessment point for all missing values.”." |
| Wright 2014  USA | N=251, 8-12 years  Overweight and obesity (from ≥85 percentile) | Comprehensive school-based program; Kids N Fitness (2 schools). Family-centered educational lifestyle program providing physical activity and dutrition education, and school-level environmental activities at the school site. 90 min weekly sessions. | | | | | General education (3 schools), participated in the standard physical activity program at ther schools and did not receive andy physical or nutritional education. | 4 mo intervention, follow-up at 12 mo | Drop-out 0-4 mo: not stated  Drop-out 0-12 mo: 24%  Reasons for drop-out: not specified | “BMI values (kg/m^2^) and associated z-scores were calculated using Epi Info software developed by CDC.”  "Individual data were analyzed under the intent-to-treat principle whereby all randomized subjects were analysed in the group to which they were randomized." |

* = studies with significant effect of intervention on change in BMI and/or BMI Z scores at one or more follow-up points (6, 12 and/or 24 months) compared to control conditions

Supplementary Table 2. Intervention components

| **Reference** | **Mean age (years)** | **Length of intervention** | **Lifestyle components** | | | **Arena** | **Group or individual intervention** | **Parent involvement** |
| --- | --- | --- | --- | --- | --- | --- | --- | --- |
|  |  |  | ***Diet*** | ***Physical activity*** | ***Behaviour*** |  |  |  |
| Backlund 2011a,b Waling 2011,2012 | 10.5 | 12 mo | x | x | x | Specialist health care | Group | Parent involvement |
| Boodai 2014 | 12.4 | 6 mo | x | x | x | Primary health care | Group | Parent involvement |
| Croker 2012 | 10.3 | 6 mo | x | x | x | Specialist health care | Individual | Parent involvement |
| Davoli 2013 | 4-7 (mean not given) | 12 mo | Aim of change | Aim of change | x  (MI) | Primary health care | Individual | Family meetings |
| DeBar 2012 | 14.1 | 5 mo | x | x | x | Primary health care | Group | Parent involvement |
| Diaz 2010 | 11.7 | 12 mo | x | Aim of change | x | Primary health care | Group | Family intervention |
| Ford 2010 | 12.6 | 12 mo | x | Aim of change | x (Mando-meter) | Specialist health care | Individual | Family intervention |
| Gong 2014 | 8.9 | 12 mo | x | x | Education | School | Group | Parent involvement |
| Gourlan 2013 | Ca 13 | 6 mo | x | Aim of change | x  (MI) | Specialist health care | Individual | Parent involvement |
| Grey 2004 | 12.6 | 4 mo | x | x | x | School | Group | Parent involvement |
| Grey 2009 | 12.7 | 4 mo | x | x | x | School | Group | Parent involvement |
| Hofsteenge 2014 | 14.5 | 6 mo | x | x | x | Specialist health care | Group | Parent involvement |
| Hughes 2008 | Ca 9 | 6 mo | x | Aim of change | x | Specialist health care | Individual | Family intervention |
| Jelalian 2010, Lloyd-Richardson 2012 | Ca 14 | 4 mo | x | x | x | Specialist health care | Group | Parent and friends involvement |
| Johnston 2007a, 2013 | 12.2 | 6 mo | x | x | x | School | Group | Parent involvement |
| Johnston 2007b, 2010 | Ca 12.5 | 6 mo | x | x | x | School | Group | Parent involvement |
| Kalarchian 2009 | 10.2 | 6 mo | x | Aim of change | x | Specialist health care | Group | Family intervention |
| Kalavainen 2007,-11,-12 | 8.1 | 6 mo | x | x | x | Specialist health care | Group | Family intervention |
| Kokkvoll 2014a,b | 10.5 | 12 mo | x | x | x | Specialist and primary health care | Group and individual | Family intervention |
| Looney 2014 | 8 | 6 mo | Aim of change | Aim of change | x | Primary health care | Individual | Family intervention |
| Maddison 2014 | 11.3 | 5 mo | - | Aim of change | x | Primary health care | Individual | Family intervention |
| Nguyen 2012, 2013 | 14.2 | 24 mo | Aim of change | Aim of change | x | Primary health care | Group | Parent involvement |
| Pakpour 2015 | 15.7 | 12 mo | Aim of change | Aim of change | X  (MI) | Specialist health care | Group | Parent involvement |
| Patrick 2013 | 14.3 | 12 mo | Aim of change | Aim of change | x | Primary health care + Internet | Group | Parent involvement |
| Pbert 2013 | 15.8 | 2 mo | Aim of change | Aim of change | x | School | Individual | Parent involvement |
| Quattrin 2012, 2014 | 4.6 | 12 mo | x | x | x | Primary health care | Group | Family intervention |
| Reinehr 2010 | 11.5 | 6 mo | x | x | x | Specialist health care | Group and individual | Family intervention |
| Savoye 2007, 2011 | Ca 12 | 12 mo | x | x | x | Specialist health care | Group | Parent involvement |
| Savoye 2014 | Ca 13 | 6 mo | x | x | x | Specialist health care | Group | Parent involvement |
| Stark 2011 | 4.7 | 6 mo | x | x | x | Specialist health care | Group and individual | Family intervention |
| Stark 2014 | 4.6 | 6 mo | x | x | x | Specialist health care | Group and individual | Family intervention |
| Steele 2012 | 11.6 | 10 weeks | x | x | x | Specialist health care | Group | Parent involvement |
| Taveras 2011 | 4.9 | 24 mo | Aim of change | Aim of change | x  (MI) | Primary health care | Individual | Family intervention |
| Van Der Baan-Slootweg 2014 | 13.9 | 6 mo | x | x | x | Specialist health care | Group | Parent involvement |
| Vos 2011, 2012 | 13.2 | 3 mo | Aim of change | Aim of change | x | Specialist health care | Group | Parent involvement |
| Wake 2009 | 7.3 | 3 mo | Aim of change | Aim of change | x | Primary health care | Individual | Parent involvement |
| Wake 2013 | 7.3 | 12 mo | Aim of change | Aim of change | x | Specialist and primary health care | Individual | Family intervention |
| Williamson 2005 | 13.2 | 6 mo | Aim of change | Aim of change | x | Internet | Individual | Parent involvement |
| Wright 2014 | 8-12 (mean not given) | 4 mo | x | x | x | School | Group | Parent involvement |

Supplementary Table 3 a. Overall GRADE judgement

| **Multicomponent lifestyle interventions compared to standard, minimal and no intervention** | | | |
| --- | --- | --- | --- |
| **Outcome**  **Time** | **Effect estimate^§^**  Mean change in: | **Number of participants**  **(studies)** | **Quality of documentation** |
| BMI  6 months | BMI was **0.99 lower** in the intervention groups (from 1,36 lower to 0,61 lower) compared to the control groups | 1591 (14) | Moderate ¹ |
| BMI  12 months | BMI was **0,67 lower** in the intervention groups (from 1,01 lower to 0,32 lower) compared to the control groups | 3238 (19) | Moderate ¹ |
| BMI  24 months | BMI was **0,96 lower** in the intervention groups (from 1,63 lower to 0,29 lower) compared to the control groups | 828 (8) | Low ^1,2^ |
|  |  |  |  |
| BMI Z score  6 months | BMI Z score was **0,12 lower** in the intervention groups (from 0,17 lower to 0,06 lower) compared to the control groups | 1607 (18) | Moderate ¹ |
| BMI Z score  12 months | BMI Z score was **0,16 lower** in the intervention groups (from 0,21 lower to 0,10 lower) compared to the control groups | 2804 (22) | Moderate ¹ |
| BMI Z score  24 months | BMI Z score was **0,16 lower** in the intervention groups (from 0,21 lower to 0,10 lower) compared to the control groups | 831 (8) | High |
| ¹High statistical heterogeneity, ^2^ Wide confidence interval | | | |
| ^§^It was not possible to calculate change in BMI / BMI Z score in intervention and control groups because we had both change score and final score in the meta-analyses | | | |
| **GRADE Working Group grades of evidence** **High quality:** We are very confident that the true effect lies close to that of the estimate of the effect **Moderate quality:** We are moderately confident in the effect estimate: The true effect is likely to be close to the estimate of the effect, but there is a possibility that it is substantially different **Low quality:** Our confidence in the effect estimate is limited: The true effect may be substantially different from the estimate of the effect **Very low quality:** We have very little confidence in the effect estimate: The true effect is likely to be substantially different from the estimate of effect | | | |

Supplementary Table 3 b. Details in the GRADE assessment

| **Quality assessment** | | | | | | | **№ of patients** | | **Effect** | | **Quality** | **Importance** |
| --- | --- | --- | --- | --- | --- | --- | --- | --- | --- | --- | --- | --- |
| **№ of studies** | **Study design** | **Risk of bias** | **Inconsistency** | **Indirectness** | **Imprecision** | **Other considerations** | **family interventions** | **usual or minimal care** | **Relative (95% CI)** | **Absolute (95% CI)** |  |  |
| BMI at 6 months (follow up: mean 6 months; assessed with: kg/m2) | | | | | | | | | | | | |
| 14 | randomised trials | not serious | serious ^1^ | not serious | not serious | none | 857 | 734 | - | MD 0.99 **lower** (1.36 lower to 0.61 lower) | ⨁⨁⨁◯ MODERATE |  |
| BMI at 12 months (follow up: mean 12 months; assessed with: kg/m2) | | | | | | | | | | | | |
| 19 | randomised trials | not serious | serious ^1^ | not serious | not serious | none | 1712 | 1526 | - | MD **0.67 lower** (1.01 lower to 0.32 lower) | ⨁⨁⨁◯ MODERATE |  |
| BMI at 24 months (follow up: mean 24 months; assessed with: kg/m2) | | | | | | | | | | | | |
| 8 | randomised trials | not serious | serious ^1^ | not serious | serious ^2^ | none | 454 | 374 | - | MD **0.96 lower** (1.63 lower to 0.29 lower) | ⨁⨁◯◯ LOW |  |
| BMI Z score at 6 months (follow up: mean 6 months; assessed with: kg/m2 SDS) | | | | | | | | | | | | |
| 18 | randomised trials | not serious | serious ^1^ | not serious | not serious | none | 846 | 761 | - | MD **0.12 lower** (0.17 lower to 0.06 lower) | ⨁⨁⨁◯ MODERATE |  |
| BMI Z score at 12 months (follow up: mean 12 months; assessed with: kg/m2 SDS) | | | | | | | | | | | | |
| 22 | randomised trials | not serious | serious ^1^ | not serious | not serious | none | 1441 | 1363 | - | MD **0.16 lower** (0.21 lower to 0.10 lower) | ⨁⨁⨁◯ MODERATE |  |
| BMI Z score at 24 months (follow up: mean 24 months; assessed with: kg/m2 SDS) | | | | | | | | | | | | |
| 8 | randomised trials | not serious | not serious | not serious | not serious | none | 452 | 379 | - | MD **0.16 lower** (0.21 lower to 0.10 lower) | ⨁⨁⨁⨁ HIGH |  |

MD – mean difference, RR – relative risk

1. High statistical heterogenety (I^2^ >50%)
2. Wide confidence interval
